# An oxidative stress-related prognostic signature for indicating the immune status of oral squamous cell carcinoma and guiding clinical treatment

**DOI:** 10.3389/fgene.2022.977902

**Published:** 2022-09-23

**Authors:** Wei Lu, Changwei Yin, Tianqi Zhang, Yihua Wu, Shengyun Huang

**Affiliations:** ^1^ Department of Oral and Maxillofacial Surgery, Shandong Provincial Hospital Affiliated to Shandong First Medical University, Jinan, Shandong, China; ^2^ Department of Oral and Maxillofacial Surgery, Shandong Provincial Hospital, Shandong University, Jinan, Shandong, China; ^3^ Department of Oral Medicine, Shandong Provincial Hospital Affiliated to Shandong First Medical University, Jinan, Shandong, China

**Keywords:** oxidative stress, gene signature, prognosis, immune status, drug sensitivity, oral squamous cell carcinoma

## Abstract

Oral squamous cell carcinoma (OSCC) is the eighth most common cancer worldwide and presents high mortality. Oxidative stress, caused by reactive oxygen species accumulation, plays a crucial role in tumorigenesis, cancer progression, and drug resistance. Nevertheless, the specific prognostic and clinical values of oxidative stress-related genes (OSGs) in OSCC remain unclear. Here, we developed an oxidative stress-related prognostic signature according to mRNA expression data from The Cancer Genome Atlas (TCGA) database and evaluated its connections with the prognosis, clinical features, immune status, immunotherapy, and drug sensitivity of OSCC through a series of bioinformatics analyses. Finally, we filtered out six prognostic OSGs to construct a prognostic signature. On the basis of both TCGA-OSCC and GSE41613 cohorts, the signature was proven to be an independent prognostic factor with high accuracy and was confirmed to be an impactful indicator for predicting the prognosis and immune status of patients with OSCC. Additionally, we found that patients with high-risk scores may obtain greater benefit from immune checkpoint therapy compared to those with low-risk scores, and the risk score presented a close interaction with the tumor microenvironment and chemotherapy sensitivity. The prognostic signature may provide a valid and robust predictive tool that could predict the prognosis and immune status and guide clinicians to develop personalized therapeutic strategies for patients with OSCC.

## Introduction

Oral squamous cell carcinoma (OSCC) accounts for most head and neck squamous cell carcinomas, and is the eighth most common cancer worldwide (Bray et al., 2018; Huang L. et al., 2021). Tobacco consumption is thought to be a major etiological factor of OSCC (Das et al., 2007). Although the progress in treatment techniques of OSCC has been notable in recent decades, the overall 5-years survival rate and recurrence rate (approximately 50%) remain disappointing (Nör and Gutkind, 2018). Therefore, it is crucial to develop an efficient and personalized therapeutic strategy for patients with OSCC. Recently, extensive efforts have been dedicated to the identification of prognostic biomarkers or signatures for OSCC based on gene expression or DNA methylation (Huang Z. et al., 2021; Huang et al., 2021b; Zou et al., 2021), whereas their specific roles in guiding personalized treatment still need to be explored in depth.

Oxidative stress is characterized by the imbalance between oxidant and antioxidant production, which contributes to an excess of reactive oxygen species (ROS) and can activate proto-oncogenes and inactivate cancer suppressor genes (Gorrini et al., 2013; Biselli-Chicote et al., 2019). ROS have been identified as a potentially critical factor in driving tumorigenesis and cancer progression (Qiu et al., 2020). Patients with OSCC have been shown to present an elevated level of oxidative stress and a compromised capacity of antioxidant defense (Choudhari et al., 2014). Oxidative stress can induce oxidative damage of DNA and protein, enhancing lipid peroxidation and antioxidant defense disorders, which, if unrepaired, can promote the formation and progression of oral cancer (Toyokuni et al., 1995; Choudhari et al., 2014). Additionally, ROS production is involved in the development of oral cancer in chewers of tobacco and areca nuts (Stich and Anders, 1989), and strictly correlates with the clinical stage in patients with advanced cancer (Mantovani et al., 2002). Furthermore, oxidative stress, as an additional metabolic feature, plays a pivotal immunoregulatory role in the tumor microenvironment (TME) (Cubillos-Ruiz et al., 2015; Maj et al., 2017). Previous studies have demonstrated that oxidative stress could not only alter the phenotype and function of myeloid dendritic cells (DCs) in the TME (Cubillos-Ruiz et al., 2015) but also control tumor Treg cell functional behavior and temper the therapeutic efficacy of immune checkpoint therapy (Maj et al., 2017). Importantly, aberrant levels of ROS can profoundly affect the tumor heterogeneity by modifying the DNA structure of cancer cells, frequently leading to chemotherapeutic resistance (de Sá Junior et al., 2017). Nevertheless, the specific roles of oxidative stress genes (OSGs) in the prognosis, immune status, and chemotherapy response of OSCC remain largely unclear.

In this study, we filtered out six prognostic OSGs to construct a predictive signature according to mRNA expression data from The Cancer Genome Atlas (TCGA) database. Then, the prognostic value of the signature and its connection with clinical features were thoroughly explored in TCGA-OSCC cohort and validated in an independent OSCC cohort GSE41613. Additionally, this signature was shown to have close connections with immune status, immunotherapy response, and chemotherapy sensitivity. Overall, our results demonstrate the potential roles of OSGs in the prognosis, immune status, and drug response of OSCC, and provide a reliable tool for predicting the prognosis of patients with OSCC and guiding clinical treatment.

## Materials and methods

### Raw data collection

The RNA-sequencing and somatic mutation data of 330 OSCC samples and 32 normal oral tissues with corresponding clinical information were acquired from the TCGA GDC portal (https://portal.gdc.cancer.gov/repository). Additionally, gene expression profiles and clinical information of 97 patients in OSCC were obtained from the GSE41613 dataset in the Gene Expression Omnibus (GEO) database (https://www.ncbi.nlm.nih.gov/geo/). A total of 1,399 protein domains related to oxidative stress were downloaded from the GeneCards database (https://www.genecards.org/), with a relevance score of ≥7 (Qiu et al., 2020). The immunohistochemistry (IHC) validation data was obtained from the Human Protein Atlas (HPA) database (https://www.proteinatlas.org/).

### Identification of differentially expressed OSGs and functional enrichment analysis

Firstly, we removed the batch effect between TCGA and GEO cohorts using “ComBat” function of “sva” R package. Then, based on the training dataset from TCGA database, we used the “limma” R package to identify the differentially expressed OSGs in OSCC samples and para-cancerous oral tissues via the Wilcoxon test, with a |log_2_fold change (FC)| > 1 and a false discovery rate (FDR) < 0.05. Gene ontology (GO) and Kyoto Encyclopedia of Genes and Genomes (KEGG) pathway enrichment analyses were used to analyze the functions and pathways associated with the differentially expressed OSGs using the R packages “clusterProfiler” and “enrichplot,” with an FDR <0.05.

### Construction of an oxidative stress-related prognostic signature

Based on the expression of differentially expressed OSGs filtered above and the survival information of patients from TCGA cohort, univariate Cox analysis of overall survival (OS) was applied to screen the prognostic OSGs via the coxph function of “survival” R package, with *p* < 0.01. Next, to minimize the risk of overfitting, we constructed a prognostic model based on the prognostic OSGs using LASSO Cox regression analysis using the R packages “survival” and “glmnet.” According to this oxidative stress-related gene signature, we calculated the risk score of each patient with OSCC on the basis of the regression coefficient and expression level of each model gene. The median risk score was regarded as a boundary to classify patients with OSCC into low-risk (LRisk) and high-risk (HRisk) groups.

### Efficacy evaluation of the gene signature

We used the R packages “survival” and “survminer” to explore the OS difference between the LRisk and HRisk groups and to draw Kaplan–Meier (KM) survival curves. Next, to evaluate the predictive accuracy and sensitivity of the gene signature, the “survival”, “survminer” and “timeROC” R packages were used to plot time-dependent receiver operating characteristic (ROC) curves and the area under the curve (AUC) values were calculated using the additive Aalen model in “timeROC” function of “timeROC” R package. The independent prognostic value of the signature was analyzed via univariate and multivariate Cox regression analyses. Additionally, we explored the correlations between the prognostic signature and clinical traits via the Wilcoxon test.

### Gene Set Enrichment Analysis in different risk groups

We performed GSEA to define the activated pathways in different risk subgroups according to the expression of the model genes. The annotated gene set “c2. cp.kegg.v7.4. symbol.gmt” was used for reference. The number of permutations was set as 1,000 and the top five pathways in each risk group were obtained to plot the enrichment results.

### TME and immunotherapy analysis

We compared the immune scores, stromal scores, and tumor purity between the LRisk and HRisk groups using the “ESTIMATE” R package. Next, the single-sample GSEA (ssGSEA) was used to estimate the infiltration levels of 16 immune cells and the enrichment scores of 13 immune-related functions in different risk groups. The different expressions of human leukocyte antigen (HLA) genes were explored in TCGA-OSCC and GEO-OSCC cohorts. Although immune checkpoint inhibitors (ICIs) can provide long-lasting clinical benefits to patients with cancer, only a fraction of patients fully respond to immunotherapy (Jiang et al., 2018). There is evidence indicating that the Tumor Immune Dysfunction and Exclusion (TIDE) algorithm and the tumor mutation burden (TMB) score can serve as predictive markers for the efficacy of ICIs (Jiang et al., 2018; Choucair et al., 2020). Therefore, to define the correlation between the prognostic signature and immunotherapy response to ICIs, we calculated the TIDE score of each patient with OSCC from the TCGA cohort online (http://tide.dfci.harvard.edu/) and analyzed the TMB score based on somatic mutation data from TCGA database. On the basis of TCGA dataset, we simultaneously calculated the RNA stemness score (RNAss) and the DNA stemness score (DNAss) based on the transcriptome data and DNA methylation data, respectively, to assess the tumor stemness of each patient with OSCC (Malta et al., 2018).

### Drug sensitivity analysis

To analyze the correlation between chemotherapeutic drug response and the risk signature, we estimated the half-maximal inhibitory concentration (IC50) of chemotherapeutic agents in TCGA cohort based on drug response data from the Cancer Genome Project (CGP) (Garnett et al., 2012) and the two largest publicly available screening efforts, the Genomics of Drug Sensitivity in Cancer (GDSC) (Yang et al., 2013) and the Cancer Therapeutics Response Portal (CTRP) (Basu et al., 2013). The CGP-derived drug response data was downloaded from the website https://osf.io/5xvsg/and processed via the “pRRophetic” package (Geeleher et al., 2014). Moreover, we retrieved GDSC2 and CTRP-derived drug response data from the website https://osf.io/c6tfx/and analyzed them using the “oncoPredict” R package (Maeser et al., 2021).

To predict potential small molecular drugs that could reverse the gene expression of high-risk patients with OSCC, the differentially expressed genes (DEGs; FDR <0.05 & |log_2_FC| > 1) between the high- and low-risk patients were uploaded to the Connectivity Map (CMap, http://www.broad.mit.edu/cmap/) and some small molecule drugs related to the risk signature were obtained online using a modified Kolmogorov–Smirnov test. A negative enrichment score indicated an inhibiting effect on the expression of high-risk genes. Finally, we set *p* < 0.05, enrichment <0, mean < −0.4 and percentage non-null ≥ 75 as the cut-off criteria (Cheng et al., 2021). The 2D chemical structures of the selected small molecule drugs were obtained from the PubChem website (https://pubchem.ncbi.nlm.nih.gov/).

### Statistical analysis

The drug sensitivity analysis via the oncoPredict R package was implemented with R software (version 4.1.1; https://www.R-project.org) and all other statistical tests were analyzed using Perl software (version 5.32; https://strawberryperl.com/) and R software (version 4.0.3). The differential analysis between two continuous variables was performed via the Wilcoxon test, and correlation analysis between two continuous variables was estimated using the Spearman’s correlation coefficient. All results were taken as statistically significant when *p* < 0.05.

## Results

### Identification of OSCC samples and OSGs

We included 459 OSCC samples in our study, including 362 from TCGA cohort (330 tumor samples and 32 normal samples) and 97 from the GSE41613 cohort. After excluding patients with no survival information and follow-up time <30 days, 322 patients in TCGA-OSCC cohort and 96 patients in GEO-OSCC cohort remained for further analyses. Detailed clinical information of the included patients is shown in Supplementary Table S1. Among 1,399 OSGs, 1,108 genes were present in both cohorts (Supplementary Table S2).

### Establishment of an oxidative stress-related prognostic signature

**FIGURE 1:**
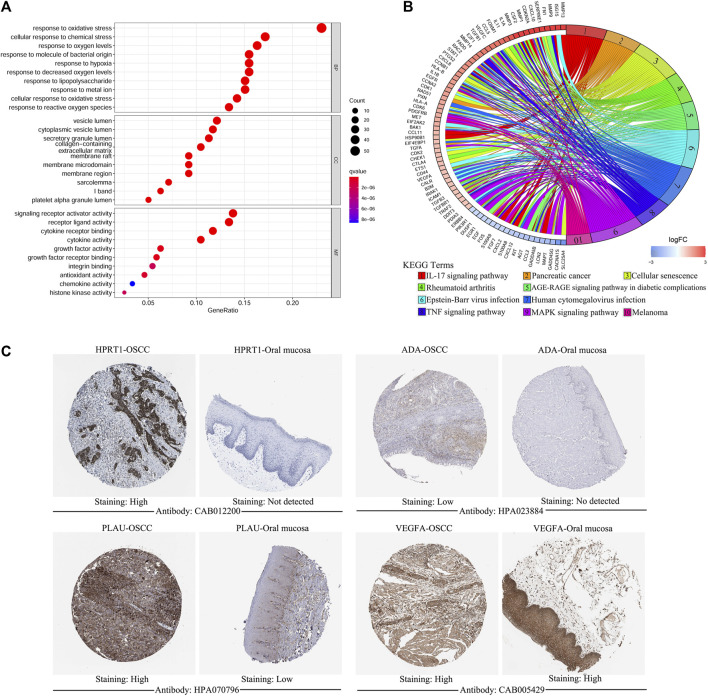
The functional enrichment analysis of oxidative stress-related differentially expressed OSGs between OSCC samples and matched adjacent normal tissues and the immunohistochemistry of signature genes from the HPA database. **(A)** GO term enrichment analysis of differentially expressed OSGs. **(B)** KEGG pathway enrichment analysis of differentially expressed OSGs. **(C)** Representative images showing the expression of HPRT1, ADA, PLAU, and VEGFA in OSCC tissues vs. normal oral cavity mucosal tissues. OSGs, Oxidative stress-related genes, OSCC, Oral squamous cell carcinoma, GO, Gene ontology, KEGG, Kyoto Encyclopedia of Genes and Genomes, HPA: Human Protein Atlas.

Of the 1,108 OSGs, 239 DEGs were obtained in OSCC tissues vs adjacent non-cancerous tissues, including 136 upregulated and 103 downregulated genes (Supplementary Table S3). As expected, GO enrichment analysis demonstrated that these differentially expressed OSGs were generally enriched in oxidative stress-related biological processes (BPs) and cytokine-related molecular functions (MFs). Furthermore, KEGG pathway enrichment analysis indicated that they were mainly associated with cancer and inflammation-related pathways (Figures 1A,B). Next, we filtered out eight differentially expressed OSGs associated with OS using univariate Cox regression analysis, among which *HPRT1*, *ADA*, *CCNA2*, *PLAU*, *IL1A*, *VEGFA*, and *CXCL8* were risk-associated OSGs (*p* < 0.01, hazard ratio [HR] > 1) and *CTLA4* was a protection-associated OSGs (*p* < 0.01, HR < 1) (Supplementary Figure S1). Finally, an oxidative stress-related prognostic model was constructed using LASSO Cox regression analysis based on TCGA-OSCC cohort and the expression of each model gene was contributing to the risk score independently with different weight according to their corresponding regression coefficient (Coef). The risk score of each patient in both TCGA-OSCC and GEO-OSCC cohorts was calculated according to the expression values of the model genes and their corresponding regression Coefs (Table 1) with the following formula: Risk score = HPRT1*Coef_*HPRT1*_ + ADA*Coef_*ADA*_ + PLAU*Coef_*PLAU*_ + CTLA4*Coef_*CTLA4*_ + VEGFA*Coef_*VEGFA*_ + CXCL8*Coef_*CXCL8*_. As a result, patients in TCGA-OSCC cohort were divided into LRisk (n = 161) and HRisk (n = 161) groups according to the median risk score, which was set as the cut-off value of risk score (0.499883). The validation cohort comprised 47 low-risk patients and 49 high-risk patients. Most signature genes (including *HPRT1*, *ADA*, *PLAU*, and *VEGFA*) were validated with IHC data from the HPA database (Figure 1C).

**TABLE 1:** The prognostic model genes and their risk coefficients.

Gene symbol	Full name	Risk coefficient
HPRT1	Hypoxanthine Phosphoribosyltransferase 1	0.0128324683391926
ADA	Adenosine Deaminase	0.0128202152397187
PLAU	Plasminogen Activator, Urokinase	0.00139422373731424
CTLA4	Cytotoxic T-Lymphocyte Associated Protein 4	-0.0960167392715765
VEGFA	Vascular Endothelial Growth Factor A	0.0196888754184647
CXCL8	C-X-C Motif Chemokine Ligand 8	0.000372634652081389

### Prognostic evaluation of the gene signature in the training and validation cohorts

**FIGURE 2:**
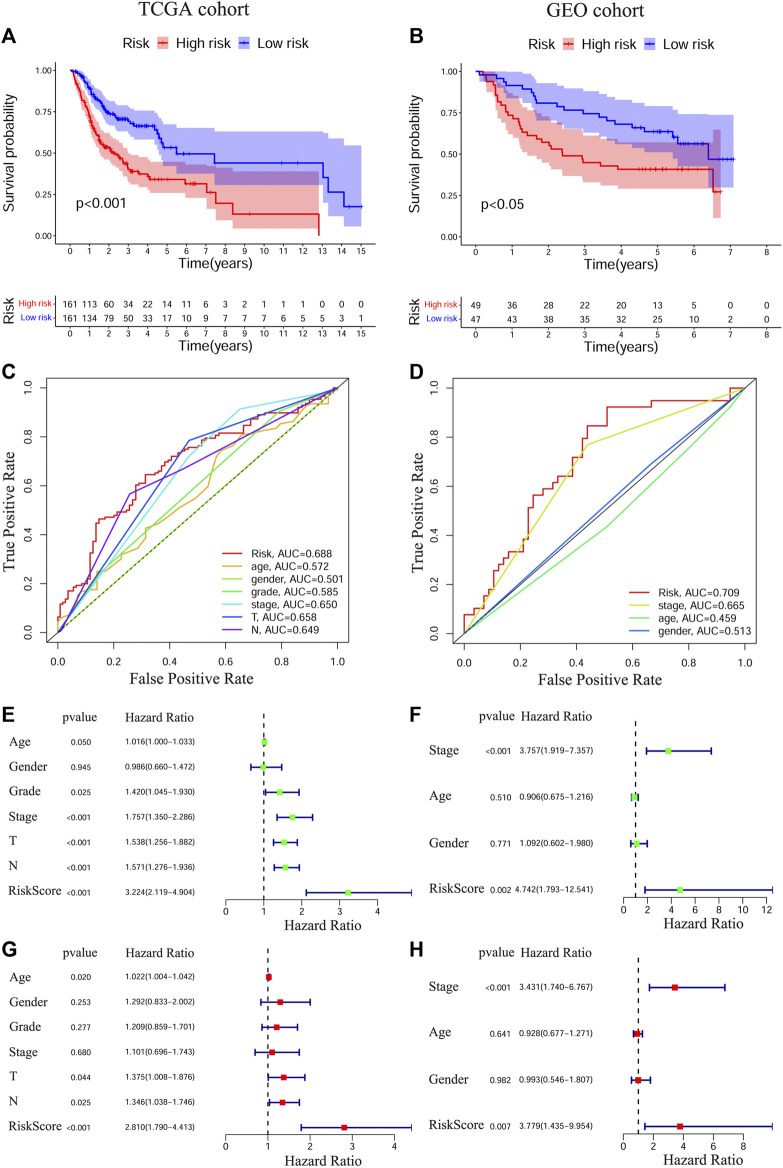
Prognostic analyses of the conducted signature in OSCC based on TCGA and GEO cohorts. The Kaplan–Meier survival curves of the prognostic signature in TCGA **(A)** and GEO **(B)** cohorts. The ROC curves and AUC values of the signature and clinical features in TCGA **(C)** and GEO **(D)** cohorts. Univariate Cox regression analyses of the signature and clinical features in TCGA **(E)** and GEO **(F)** cohorts. Multivariate Cox regression analyses of the signature and clinical features in TCGA **(G)** and GEO **(H)** cohorts. OSCC, Oral squamous cell carcinoma, TCGA, The Cancer Genome Atlas, GEO, The Gene Expression Omnibus, ROC, Receiver operating characteristic, AUC, Area under curve.

To test the prognostic values of the gene signature, we performed KM survival analysis, time-dependent ROC exploration, and univariate and multivariate Cox regression analyses in both the training and validation cohorts. As expected, patients in the HRisk group had a significantly worse OS than those in the LRisk group according to the KM survival curves of the two cohorts (TCGA: Figure 2A, *p* < 0.001; GEO: Figure 2B, *p* < 0.05). The accuracy of the gene signature in survival prediction was explored with time-dependent ROC curves and their corresponding AUC values (Figures 2C,D, Supplementary Figures S1B,C). The AUC values of the risk score reached 0.688 in TCGA-OSCC cohort and 0.709 in GEO-OSCC cohort at 3 years, which were both higher than those of the clinical variables (Figures 2C,D). To test the independent prognostic value of the model, we generated univariate and multivariate Cox regression analyses of OS. On the basis of univariate Cox analysis, the risk score in both the training and validation cohorts was significantly associated with OS (TCGA: Figure 2E, HR = 3.224, 95% confidence interval [CI] = 2.119–4.904, *p* < 0.001; GEO: Figure 2F, HR = 4.724, 95% CI = 1.793–12.541, *p* < 0.01). After adjusting for other confounding clinical factors using multivariate Cox analysis, the risk score was found to be an independent prognostic predictor of patients with OSCC (TCGA: Figure 2G, HR = 2.81, 95% CI = 1.79–4.413, *p* < 0.001; GEO: Figure 2H, HR = 3.779, 95% CI = 1.435–9.954, *p* < 0.01).

### Expression levels and clinical features underlying the prognostic signature

**FIGURE 3:**
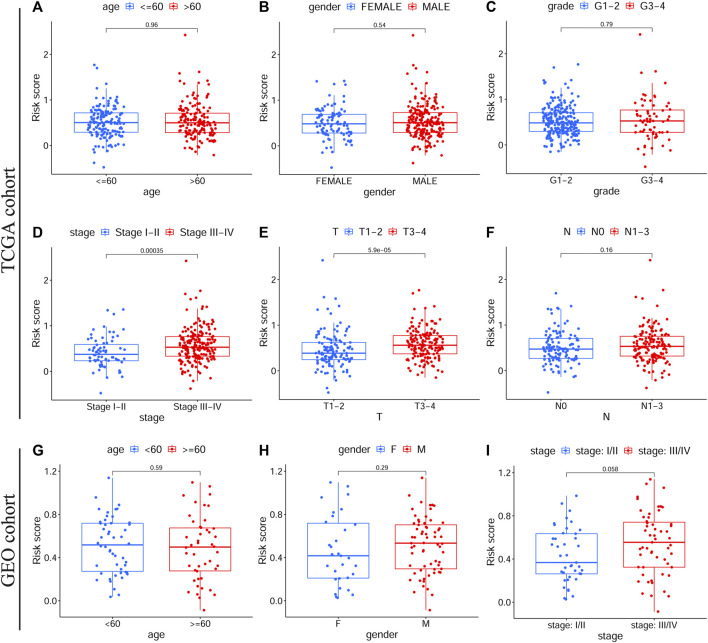
The correlations between clinical features and the prognostic signature. Different risk scores in OSCC patients with different age **(A)**, gender **(B)**, grade **(C)**, clinical stage **(D)**, T stage **(E)** and N stage **(F)** according to TCGA cohort. Different risk scores in OSCC patients with different age **(G)**, gender **(H)**, and clinical stage **(I)** on basis of the validation cohort. OSCC, Oral squamous cell carcinoma, TCGA, The Cancer Genome Atlas.

Among patients in the training and validation cohorts, we compared the expression levels of all model genes between the HRisk and LRisk groups. As expected, the expression levels of risk-associated model genes were all significantly upregulated in the HRisk group, while the protection-associated *CTLA4* was notably overexpressed in the LRisk group on the basis of both TCGA-OSCC and GEO-OSCC cohorts (Supplementary Figure S2, all *p* < 0.001). Based on the optimal cut-off expression value, we performed survival analysis of each model gene using the “survival” and “survminer” R packages, which demonstrated that the expression levels of all model genes were significantly correlated with OS in TCGA-OSCC cohort Supplementary Figures S3A–F, *p* < 0.01), and similarly, except for *VEGFA* (Supplementary Figure S3L, *p* = 0.072), the expression of the other model genes was significantly correlated with OS in the GEO-OSCC cohort (Supplementary Figures S3G,F, *p* < 0.05). However, patients with lower expression of *VEGFA* had longer OS in the GEO-OSCC cohort, although the *p*-value was not significant, possibly due to the small number of patients with lower expression of *VEGFA* (n = 13). The above results suggest that the prognostic model genes could serve as potential therapeutic targets for patients with OSCC. According to KM survival analyses, we found that clinical stage, T stage, and N stage had significant prognostic values in OSCC. As shown in Supplementary Figure S4, patients with clinical stage I–II, T_1–2_, or N_0_ had significantly better OS than those with stage III–IV (*p* < 0.001), T_3–4_ (*p* < 0.001), or N_1–3_ (*p* < 0.01) in TCGA-OSCC cohort. Additionally, survival analyses in the GEO-OSCC cohort confirmed that the stage I–II group was significantly correlated with better OS compared to the stage III–IV group (*p* < 0.001) (there were no data on the clinical T and N stages of OSCC in the GEO cohort). Next, by analyzing the correlation between the clinical features and risk score via the Wilcoxon test, we confirmed that patients with stage III–IV or T_3–4_ were significantly related to a higher risk score, while patients with stage I–II or T_1–2_ understandably had a lower risk score in TCGA-OSCC cohort (Figures 3D,E, *p* < 0.001), which might determine the worse OS of patients in HRisk group. Moreover, patients with stage III–IV still presented a higher mean risk score than those with stage I–II in the GEO-OSCC cohort, although there was no significant difference in the risk score between patients with different clinical stages (Figure 3I, *p* = 0.058). Therefore, the identified signature could be involved in the occurrence and development of OSCC.

### Functional enrichment analysis in the HRisk and LRisk groups

**FIGURE 4:**
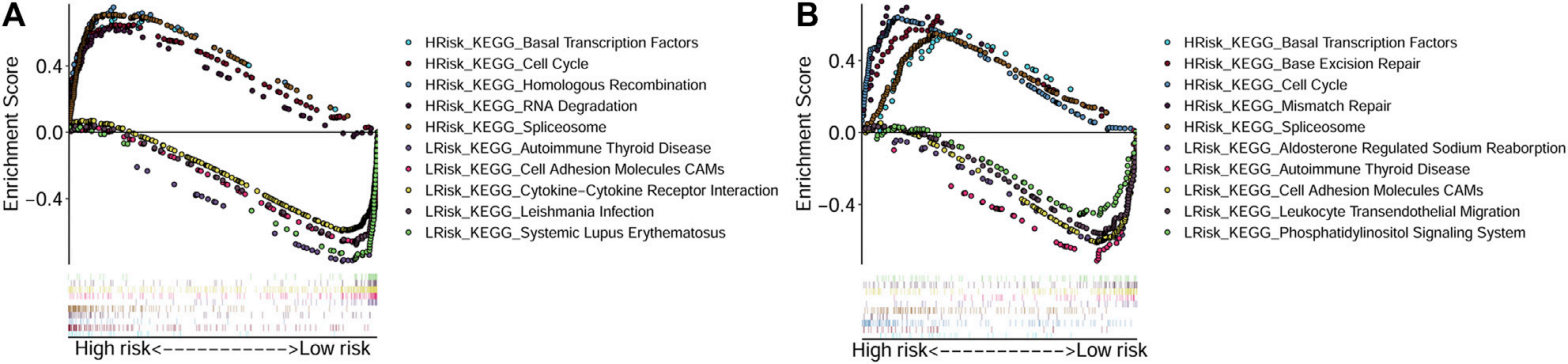
Gene Set Enrichment Analyses based on the conducted signature. **(A)** Active pathways in the HRisk and LRisk groups in TCGA cohort. **(B)** Active pathways in the HRisk and LRisk groups in GEO cohort. GEO: The Gene Expression Omnibus, TCGA, The Cancer Genome Atlas, HRisk, high-risk, LRisk, low-risk.

To explore the main functions enriched in different risk subgroups, we performed GSEA in both TCGA-OSCC and GEO-OSCC cohorts. The results indicated that pathways that were active in the HRisk group were mainly cell cycle-related, while those enriched in the LRisk group were mostly related to autoimmunity and cell adhesion in both the training and validation cohorts. The detailed results of GSEA are shown in Figure 4.

### TME and immunotherapy response analysis

**FIGURE 5:**
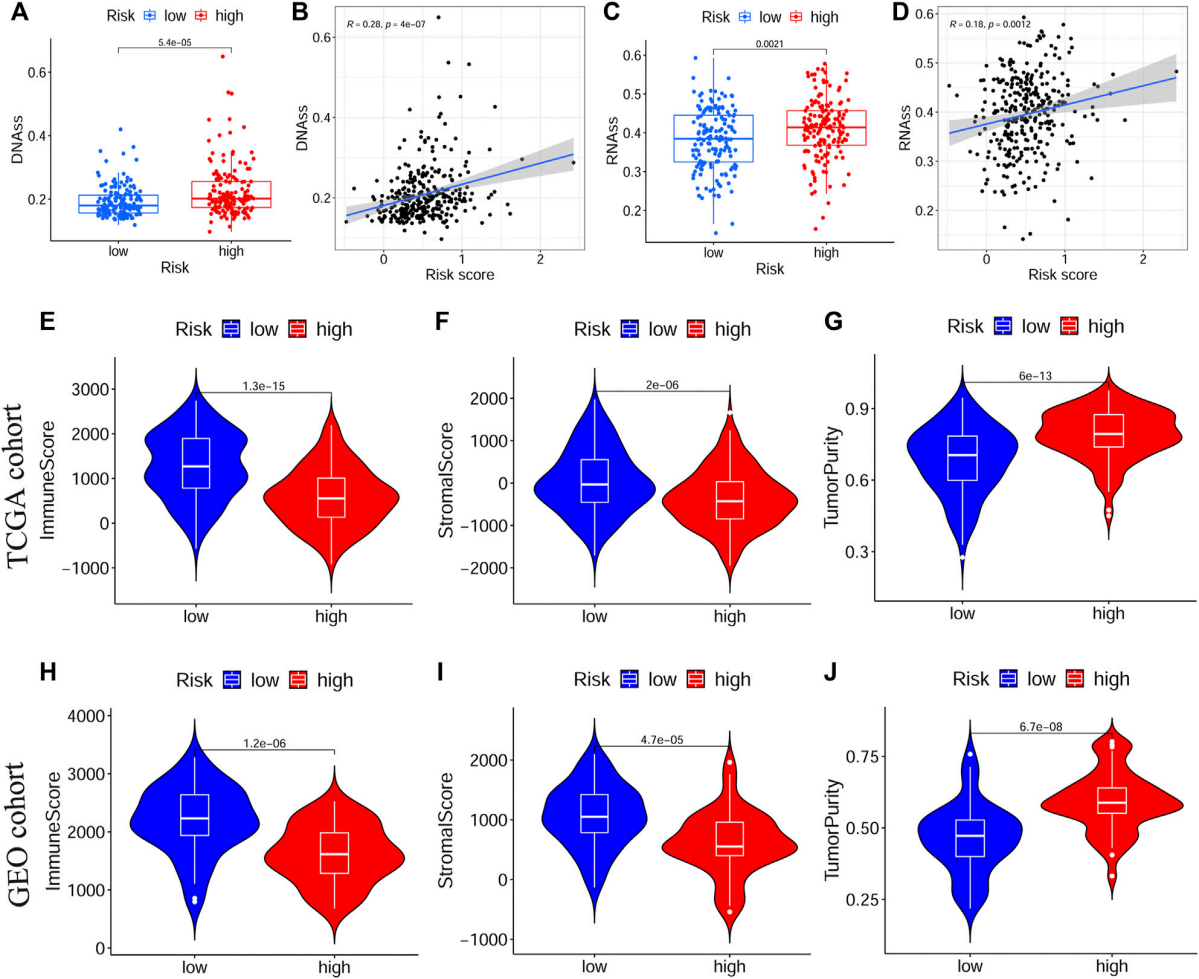
The tumor stemness and immune microenvironment between different risk subgroups. **(A,B)** Comparison of DNAss and RNAss in different risk subgroups. **(C,D)** The relationship between risk score and DNAss or RNAss based on TCGA cohort. **(E–G)** The immune score, stromal score and tumor purity between different risk groups in TCGA cohort. **(H–J)** The immune score, stromal score and tumor purity between different risk groups in GEO cohort. TCGA, The Cancer Genome Atlas, GEO, The Gene Expression Omnibus.

**FIGURE 6:**
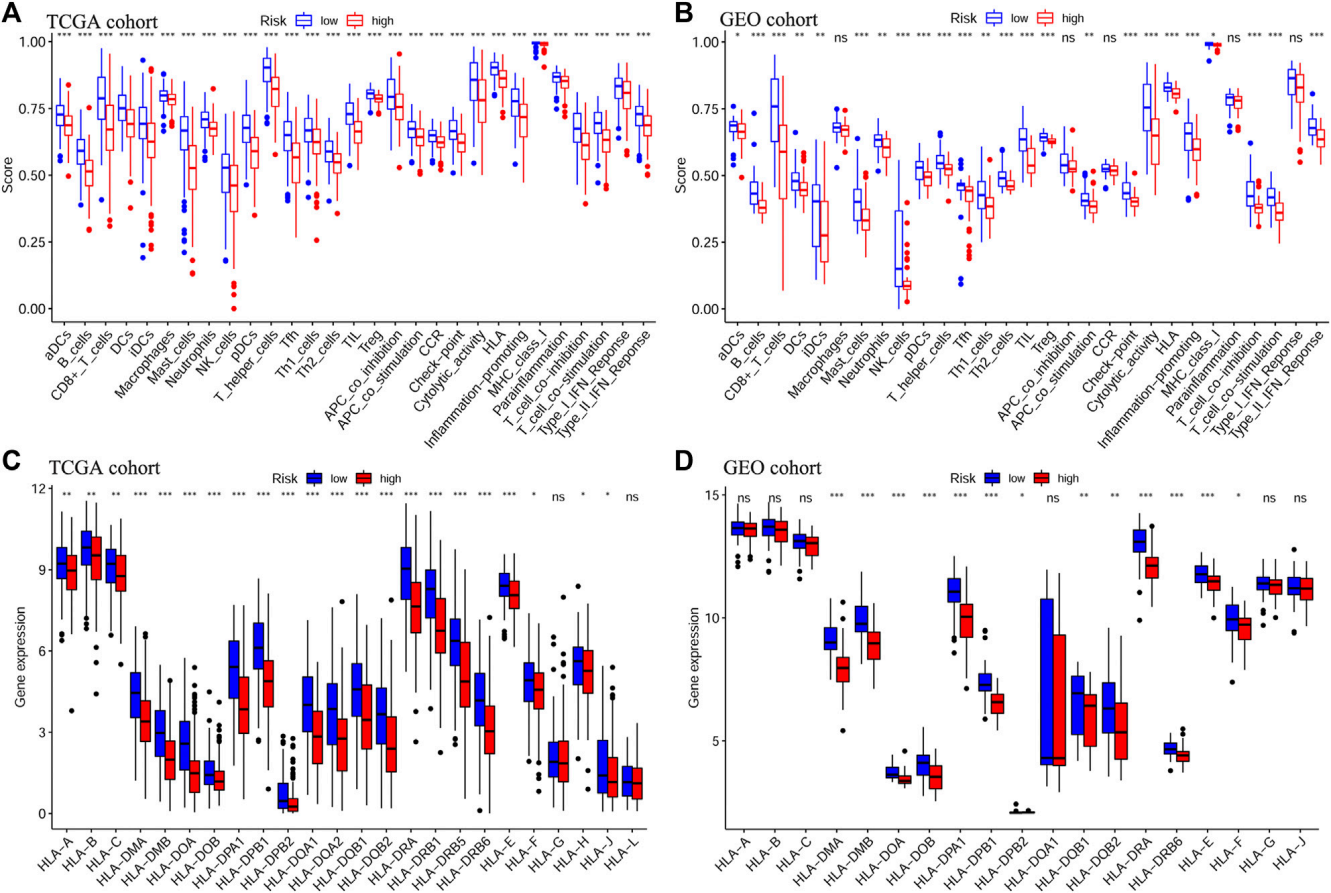
Analyses of immune cells and immune functions of the prognostic signature. Evaluation of infiltrating scores of 16 immune cells and activity of 13 immune-related pathways in different risk groups via ssGSEA according to TCGA cohort **(A)** and GEO cohort **(B)**. Differentially expressed analysis of 24 HLA genes based on TCGA cohort **(C)** and GEO cohort **(D)**. The *p* values were showed as: *p < 0.05; **p < 0.01; ***p < 0.001. ssGSEA: single-sample Gene Set Enrichment Analysis, TCGA, The Cancer Genome Atlas, GEO, The Gene Expression Omnibus, HLA, Human leukocyte antigen.

Next, we evaluated the correlation between the prognostic signature and the TME in TCGA-OSCC and GEO-OSCC cohorts. The ESTIMATE results demonstrated that patients in the LRisk group presented notably higher immune and stromal scores, but a lower level of tumor purity compared to those in the HRisk group (all *p* < 0.001, Figures 5E–J). Consistently, the risk score negatively correlated with both the immune score and stromal score, whereas it showed a positive relationship with the level of tumor purity (all *p* < 0.001, Supplementary Figure S5). Meanwhile, the tumor stemness of the patients with OSCC was estimated using DNAss and RNAss based on the TCGA dataset. Both DNAss (*p* < 0.001, Figure 5A) and RNAss (*p* < 0.01, Figure 5C) were obviously higher in the HRisk group compared to those in the LRisk group. Similarly, both DNAss (*p* < 0.001, Figure 5B) and RNAss (*p* < 0.01, Figure 5D) were positively related to the risk score. We then estimated the enrichment levels of immune cells and immune-related functions in different risk groups using ssGSEA to accurately assess their immune status. The LRisk group displayed significantly higher infiltration levels of some innate immune cells, including mast cells, neutrophils, natural killer (NK) cells, DCs, immature DCs (iDCs), activated DCs (aDCs), and plasmacytoid DCs (pDCs), as well as adaptive immune cells, including B cells, CD8^+^ T cells, helper T cells, regulatory T (Treg) cells, T helper 1 (Th1) cells, Th2 cells, T follicular helper cells (Tfh), and tumor-infiltrating lymphocytes (TILs). The LRisk group also presented higher enrichment scores of some immune-related functions, including cytolytic activity, checkpoint, promoting inflammation, type II IFN response, T cell co-stimulation, and HLA (Figures 6A,B). KM survival curves based on the optimal cut-off value demonstrated that immune cells (including DCs, aDCs, iDCs, mast cells, neutrophils, NK cells, B cells, and helper T and Treg cells) and immune functions (including cytolytic activity, checkpoint, type II IFN response, T cell co-stimulation, and HLA) showed a beneficial effect on the prognosis of patients with OSCC according to both the training and validation cohorts (all *p* < 0.05, Supplementary Figure S6), which may result in the better prognosis of the LRisk group. Considering HLA markers play a crucial role in anti-tumor immunity by driving antigen presentation (Anderson et al., 2021), we analyzed the differential expression of 24 HLA genes between two risk groups. The results demonstrated that the LRisk group had higher expression levels of most HLA genes according to both TCGA-OSCC and GEO-OSCC cohorts (Figures 6C,D). Overall, patients in the LRisk group showed more active immune activity, which may explain their better prognosis.

**FIGURE 7:**
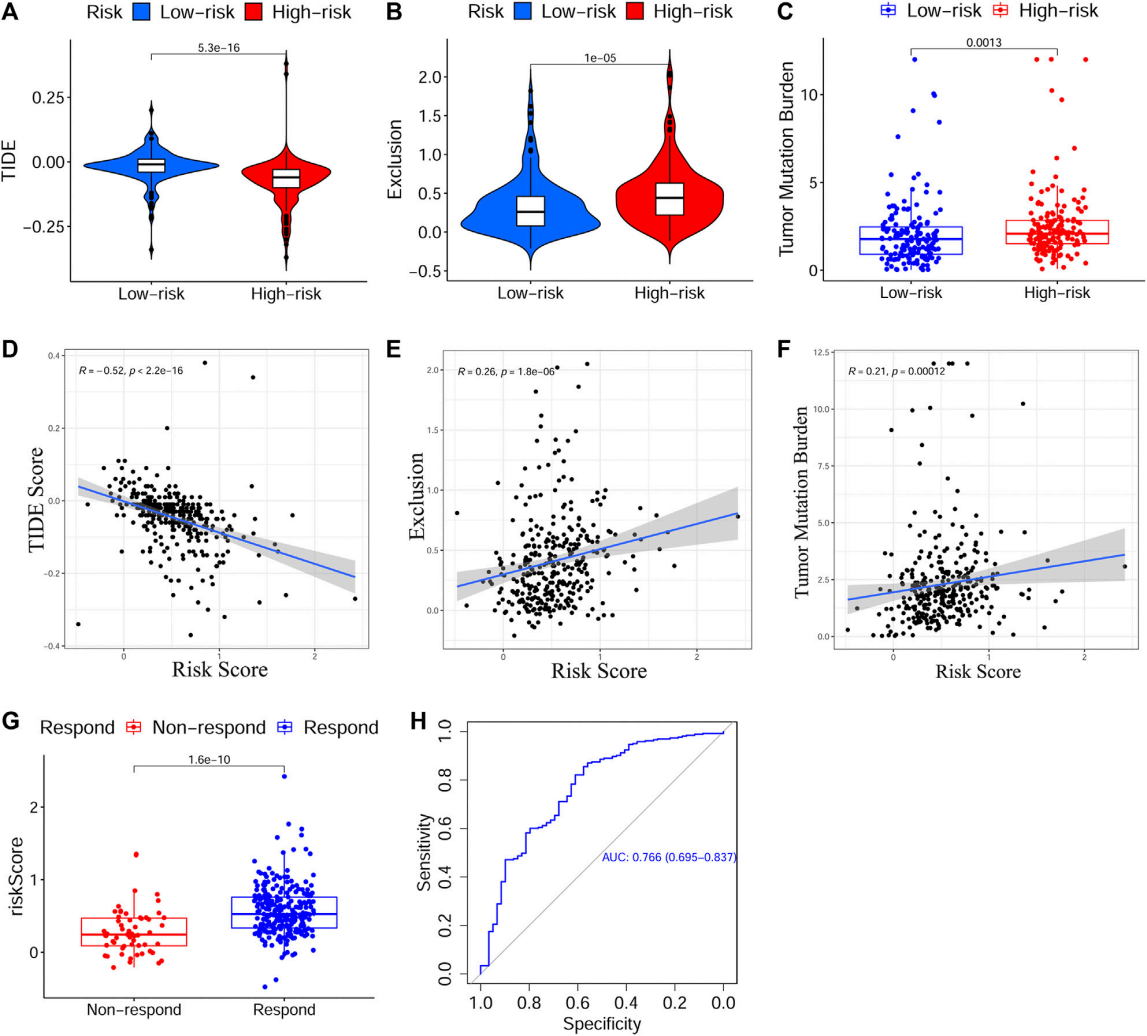
Evaluation of the predictive effect of the prognostic signature on ICI treatment response. **(A–C)** The scores of TIDE, immune exclusion and TMB in different risk subgroups. **(D–F)** The relationship between the risk score and the score of TIDE, immune exclusion and TMB. **(G)** Predicted ICI treatment responses in different risk subgroups based on TCGA cohort using TIDE algorithm. **(H)** The ROC curve and AUC value to estimate the accuracy of the signature for predicting ICI treatment response. ICI, Immune checkpoint inhibitor, TIDE, Tumor Immune Dysfunction and Exclusion, TMB, Tumor Mutation Burden, TCGA, The Cancer Genome Atlas, ROC, Receiver operating characteristic, AUC, Area under curve.

Next, to evaluate the predictive effect of the risk signature on the immunotherapy response, we explored the relationship between the risk score and the TIDE/TMB score. On the basis of the TIDE algorithm, the results revealed that patients in the HRisk group presented prominently lower TIDE scores (*p* < 0.001, Figure 7A), higher immune exclusion scores (*p* < 0.001, Figure 7B), and greater immunotherapy response (*p* < 0.001, Figure 7G). Meanwhile, the risk score was positively associated with the TIDE score (*p* < 0.001, Figure 7D) and negatively related to the immune exclusion score (*p* < 0.001, Figure 7E). To estimate the sensitivity of the risk score for predicting an immunotherapy response, a ROC curve was plotted using “pROC” R package and the AUC value was 0.766 (95% CI: 0.695–0.837, Figure 7H). Moreover, we found that the TMB score in the HRisk group was notably higher than that in the LRisk group (*p* < 0.01, Figure 7C), and the risk score had a positive relationship with the TMB score (*p* < 0.001, Figure 7F). In view of the above results, the risk signature is an appropriate predicting indicator of ICI treatment with high sensitivity.

### Selecting appropriate chemotherapy drugs and uncovering potential small molecular drugs

**FIGURE 8:**
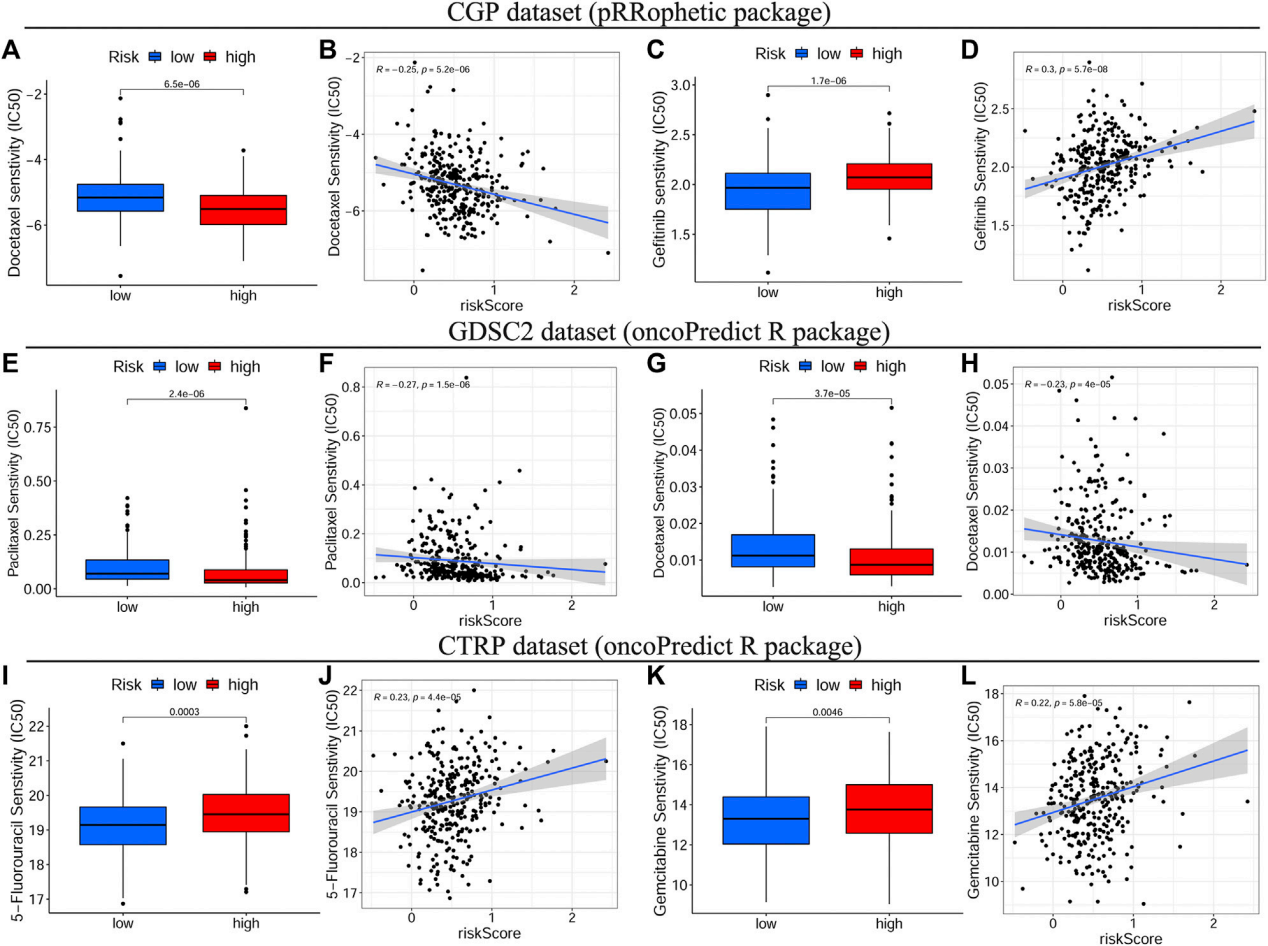
Drug sensitivity analyses on basis of TCGA cohort. The IC50 of some selected chemotherapeutic drugs, including CGP/GDSC2-derived Docetaxel **(A–G)**, CGP-derived Gefitinib **(C)**, GDSC2-derived Paclitaxel **(E)** and CTRP-derived drugs, 5-Fluorouracil and Gemcitabine **(I,K)** in different risk subgroups. The correlations between the risk score and some selected chemotherapeutic drugs, including CGP/GDSC2-derived Docetaxel **(B–H)**, CGP-derived Gefitinib **(D)**, GDSC2-derived Paclitaxel **(F)** and CTRP-derived drugs, 5-Fluorouracil and Gemcitabine **(J,L)**. TCGA, The Cancer Genome Atlas, IC50, Half-maximal inhibitory concentration, CGP, the Cancer Genome Project, GDSC, Genomics of Drug Sensitivity in Cancer, CTRP, Cancer Therapeutics Response Portal.

To identify the relationship between drug response and the risk signature, we evaluated the differential drug response between HRisk and LRisk groups (*p* < 0.05 was considered significant) and performed the Spearman correlation test between the IC50 of selected chemotherapy drugs and the risk score (|R| > 0.2 and *p* < 0.05 were set as the threshold values). Eight common OSCC chemotherapy drugs (Cisplatin, Paclitaxel, Cytarabine, Docetaxel, Doxorubicin, Gemcitabine, Methotrexate, and 5-Fluorouracil) were selected for predicting the drug response on the basis of National Comprehensive Cancer Network (NCCN) guidelines Version 2.2021. Additionally, Gefitinib, an orally active selective EGFR (epidermal growth factor receptor) inhibitor, was explored based on previous studies (Brown et al., 2010; Tang et al., 2019). The detailed results of all selected drugs are shown in Supplementary Figure S7. Consequently, patients with low-risk scores showed a more sensitive response to CTRP-derived drugs (5-Fluorouracil and Gemcitabine, Figures 8I–L) and CGP-derived Gefitinib (Figures 8C,D), while patients with high-risk scores were more sensitive to GDSC2-derived Paclitaxel (Figures 8E,F) and CGP/GDSC2-derived Docetaxel (Figures 8A,B,G,H).

To uncover novel drugs for treating patients with OSCC in the HRisk group, we analyzed the DEGs between the LRisk and HRisk groups using the CMap algorithm. CMap analysis was used to screen out 10 small molecule drugs (including etifenin, cortisone, sulfaguanidine, cyclopenthiazide, alcuronium chloride, xylometazoline, dextromethorphan, AH-6809, methylprednisolone, and oxybuprocaine) (Table 2), which were considered likely to reverse the expression of high risk-related genes and may be novel drugs showing anti-tumor effects in HRisk patients with OSCC. Furthermore, we obtained 2D chemical structures of the selected small molecule candidates online to facilitate future studies, which are shown in Supplementary Figure S8.

**TABLE 2:** Potential small molecular drugs targeting high-risk OSCC patients.

Drug name	enrichment	P Value	mean	percent non-null
etifenin	−0.907	0.0001	−0.634	100
cortisone	−0.931	0.0005	−0.648	100
sulfaguanidine	−0.761	0.00144	−0.424	80
cyclopenthiazide	−0.826	0.00175	−0.413	75
alcuronium chloride	−0.918	0.01368	−0.579	100
xylometazoline	−0.677	0.02425	−0.456	75
dextromethorphan	−0.664	0.02863	−0.511	75
AH-6809	−0.871	0.03314	−0.588	100
methylprednisolone	−0.65	0.03537	−0.435	75
oxybuprocaine	−0.626	0.04852	−0.513	75

OSCC, oral squamous cell carcinoma.

## Discussion

Oxidative stress, caused by ROS accumulation, plays a crucial role in cancer cells throughout the initiation, progression, metastasis, recurrence, and therapy of multiple tumors (Hayes et al., 2020). Accumulating evidence shows that the ROS levels not only correlate with tumor growth but also affect both the TME and the sensitivity of cancer cells to chemotherapeutic agents (Sosa et al., 2013; de Sá Junior et al., 2017). Additionally, oxidative stress-related gene signatures have been identified to be a reliable and efficient tool to predict the prognosis and progression of cancers (Qiu et al., 2020; Wu et al., 2021). Considering the above views, OSGs may represent valuable biomarkers for predicting the prognosis, immune status, and drug sensitivity of cancers and thus help clinicians define individual treatment plans for patients. However, the predictive effect of OSGs in OSCC remains unclear and is yet to be thoroughly investigated. Hence, in this study, we first filtered out 239 aberrantly expressed OSGs in OSCC and explored their potential functional pathways in OSCC. Next, we selected eight prognostic OSGs, according to which an oxidative stress-related signature with six OSGs was conducted. Moreover, the main functions active in different subgroups classified by the risk signature were identified using GSEA analysis. Finally, to further define the specific roles of OSGs in OSCC, we deeply evaluated the correlations between the oxidative stress-related signature and the prognosis, clinical features, immune status, immunotherapy, and drug sensitivity of OSCC.

The oxidative stress-related prediction signature comprised five risk-associated OSGs (*HPRT1*, *ADA*, *PLAU*, *VEGFA*, and *CXCL8*) and one protection-associated OSG (*CTLA4*), which were verified to be prognostic indicators in OSCC via univariate Cox regression and KM survival analyses. Moreover, these five risk OSGs were significantly upregulated in the HRisk group, while *CTLA4*, as a protection gene, was notably overexpressed in the LRisk group. *HPRT1*, a salvage enzyme involved in nucleotide production and recycling in cell cycle modulation, has been shown to promote proliferation and metastasis of head and neck squamous cell carcinoma (HNSCC) through direct interaction with STAT3 and has been implicated as a promising prognostic indicator and potential therapeutic target for HNSCC (Wang et al., 2021b). *ADA*, a housekeeping enzyme crucial in purine metabolism, makes a certain contribution to the regulation of inflammatory reactions and immune status (Bagheri et al., 2019), and its inhibitor has been shown to be obviously associated with reduced tumor size and decreased aggressiveness of cancer cells (Kutryb-Zajac et al., 2018; Bagheri et al., 2019). Additionally, Wang et al. reported that pre-operative serum ADA levels could be a reliable independent prognostic predictor of OSCC (Zhu et al., 2019). Previous studies demonstrated that the upregulated expression of *PLAU* (uPA), *VEGFA*, and *CXCL8* (IL8) could promote the occurrence and progression of OSCC and might be important prognostic factors for patients with OSCC (Hundsdorfer et al., 2005; Sales et al., 2016; Magnussen et al., 2017; de Matos et al., 2019; Reyimu et al., 2021). The immune checkpoint inhibitory receptor CTLA4 has also been identified as a potential therapeutic target for OSCC (Bundela et al., 2014) and can enhance the therapeutic efficacy of anti-PD-1 immunotherapy in patients with HPV^+^ OSCC (Dorta-Estremera et al., 2019). Combining the results of the current and previous studies, we believe that these six OSGs could serve as reliable prognostic biomarkers and provide potential treatment targets for OSCC.

The prediction signature, composed of six prognostic OSGs with different weighting coefficients, was verified to be an effective prognostic indicator of OSCC according to the training and validation cohorts using univariate Cox regression and KM survival analyses. Additionally, patients with OSCC in the HRisk group had significantly shorter OS than those in the LRisk group. Multivariate Cox regression analysis indicated that the six-gene prediction signature was an independent prognostic predictor of OS of patients with OSCC. Furthermore, the results of ROC curves and AUC values validated the high prediction accuracy of this signature. In terms of the relationship between the risk score and clinical features, patients with tumor stage III–IV or T_3–4_ were significantly associated with a higher risk score, which suggested that the risk score increases with the progression of OSCC, showing the poorer prognosis of patients with the higher risk score.

We next performed GSEA analysis in both the training and validation cohorts to define the specific signal pathways involved in the oxidative stress-related signature. The results demonstrated that cell cycle-related pathways such as the cell cycle, spliceosome, and base excision repair were apparently activated in the HRisk group. Moreover, oxidative stress-mediated ROS production exerts a key influence on cell cycle dysregulation by incorporating phosphorylation, ubiquitination, and receptor activation, which can contribute to aberrant cell proliferation and promote tumor progression (Verbon et al., 2012; Ahmad et al., 2020). Therefore, the activated cell cycle-related pathways may relate to the poor prognosis of patients with OSCC in the HRisk group. Meanwhile, autoimmunity-related pathways (i.e., autoimmune thyroid disease and systemic lupus erythematosus) were enriched in the LRisk group, illustrating that the patients with OSCC in the LRisk group might present an active immune state.

Growing evidences suggest that the TME plays a critical role in carcinogenesis, tumor progression, and survival, among which the immune microenvironment serves as a determinative factor (Hinshaw and Shevde, 2019). Thus, we next explored the relationship between the constructed signature and the immune status of OSCC. According to the ESTIMATE algorithm, a higher risk score was notably correlated with a lower immune score based on TCGA-OSCC and GEO-OSCC cohorts, suggesting that the high-risk score indicated an immune-suppressive state of patients with OSCC. Additionally, patients in the HRisk group displayed significantly higher tumor immune evasion scores compared to low-risk patients, and the immune evasion score showed a positive correlation with the risk score on the basis of the TIDE results in TCGA-OSCC cohort, further demonstrating the poor prognosis and immune-suppressive state of patients in the HRisk group. When we estimated the correlation between the prognostic signature and immune functions using ssGSEA, we found that compared to patients in the HRisk group, those in the LRisk group presented higher enrichment scores of cytolytic activity, promoting inflammation, the type II IFN response, and HLA. Of note, the abovementioned immune functions were all positively associated with a favorable prognosis in patients with OSCC according to both the training and validation cohorts via KM survival analysis, which contributes to the beneficial prognosis of low-risk patients. Rooney et al. reported that the cytolytic activity was associated with a modest but significant pan-cancer survival benefit and was connected to counter-regulatory immune responses (Rooney et al., 2015). Consistently, high cytolytic activity in tumor-free tongue tissue conferred improved prognosis in patients with tongue squamous cell carcinoma (Gu et al., 2019). Type II IFN (IFNγ) is thought to play a crucial role in cancer immunosurveillance, with the ability to promote anti-tumor immunity by increasing tumor immunogenicity (Dunn et al., 2006). Naturally, HLA markers are essential for antigen presentation and display a pivotal role in anti-tumor immunity by enhancing immunosurveillance and preventing immune escape. Additionally, high HLA class I expression in OSCC shows a significantly positive connection with a better prognosis (Koike et al., 2020; Anderson et al., 2021). Besides, it is worth emphasizing that various immune cells in the TME participate in anti-tumor immune responses as main components. Hence, we simultaneously calculated the enrichment level of immune cells in different risk subgroups using ssGSEA. The results showed that patients in the LRisk group displayed significantly higher infiltration of DCs, NK cells, mast cells, neutrophils, B cells, helper T cells, and Treg cells, each of which had a positive relationship with favorable prognosis in patients with OSCC. As the most potent antigen-presenting cells, DCs underpin the successful generation of anti-tumor immune responses by initiating and regulating innate and adaptive immune responses in the TME, and thus, targeting DCs is a promising strategy to improve the efficacy of current immunotherapies (Verneau et al., 2020). NK cells, with the potent ability to kill tumor cells, induce remodeling of the oral TME via IFN-γ and TNF-α, as well as prevent tumor growth and metastasis (Jewett et al., 2018). Furthermore, both CD103^+^ DCs and activated NK cells have been shown to have a favorable prognosis in OSCC (Hadler-Olsen and Wirsing, 2019; Xiao et al., 2019). Interestingly, Tregs were significantly connected to an improved prognosis of OSCC in our study, despite being recognized as immunosuppressive cells in numerous cancers. Among patients with OSCC, a high level of infiltrated Tregs has been proven to be notably associated with a lower frequency of lymph node metastasis and prolonged OS (Bron et al., 2013). Moreover, Hanakawa et al. suggested that Tregs in the TME may prevent tumor cell invasion and metastasis by inhibiting inflammatory processes (Hanakawa et al., 2014). In brief, the immune-related results in our study expectedly cohere with those in previous studies and the improved prognosis of patients in the LRisk group may be a result of the active immune state and increasing anti-tumor immune responses in these patients.

In addition to the TME, as the central component of cancers, tumor cells naturally play a vital role in tumorigenesis. Tumor cells present distinct phenotypic states with different functional attributes, among which, cancer stem cells (CSCs) possess the principal properties of self-renewal, clonal tumor initiation capacity, and clonal long-term repopulation potential (Plaks et al., 2015). From this perspective, CSCs can facilitate the initiation and progression of tumors, which may induce drug resistance (Plaks et al., 2015; Malta et al., 2018). Thus, we evaluated the correlation between the prognostic signature and the tumor stemness of OSCC. Based on DNAss and RNAss, patients with OSCC in the HRisk group presented higher tumor stemness than those in the LRisk group, and the risk score showed a positive connection with tumor stemness. These results suggested that this oxidative stress-related signature could predict tumor progression and invasion for patients with OSCC and simultaneously explain the poor prognosis of high-risk patients.

Presently, personalized medicine, concentrating on designing specific therapeutics that are best suited for an individual patient based on genome information, is considered to be the future direction in oncotherapy (Jackson and Chester, 2015). To verify whether the prognostic signature could guide clinicians to develop an effective personalized treatment decision for patients with OSCC, we thoroughly investigated the correlation between the constructed signature and response to immunotherapy or chemotherapy. Immunotherapy, particularly ICI treatment, offers a reliable alternative or adjunctive therapy to conventional therapies for refractory patients with OSCC (Dorta-Estremera et al., 2019). The TIDE score has been identified as a potent biomarker to predict the tumor response to anti-PD1 or anti-CTLA4 (Jiang et al., 2018), while the TMB score has also been validated as an available tool to help oncologists select patients who may benefit from ICIs (Choucair et al., 2020). Encouragingly, the signature in our study was not only closely correlated with both TIDE and TMB scores but was also efficient in predicting the immunotherapy response with high accuracy (AUC = 0.766). Next, we comprehensively evaluated the efficacy of chemotherapy drugs in different risk subgroups and their relationship to the risk score on the basis of three public drug sensitivity databases (CGP, GDSC, and CTRP) (Garnett et al., 2012; Basu et al., 2013; Yang et al., 2013). The IC50 values of eight common chemotherapy drugs for OSCC (Cisplatin, Paclitaxel, Cytarabine, Docetaxel, Doxorubicin, Gemcitabine, Methotrexate, and 5-Fluorouracil), on the basis of the NCCN guidelines Version 2.2021, and one novel anti-cancer drug, Gefitinib, based on previous studies, were calculated and estimated. Consequently, we found that patients with low-risk scores were more sensitive to 5-Fluorouracil, Gemcitabine, and Gefitinib, while those with high-risk scores showed more sensitive responses to paclitaxel and docetaxel, which indicated that the prognostic signature could facilitate personalized chemotherapy decisions. Furthermore, 5-Fluorouracil, Gemcitabine, and Gefitinib generate ROS, which can cause cancer cell death by inducing oxidative damage, whereas CSCs can increase cellular adaptive responses to ROS to result in chemoresistance (Okon et al., 2015; Blondy et al., 2020; Xue et al., 2020). As mentioned above, OSCC patients with high-risk scores presented notably elevated tumor stemness, which may explain their higher resistance to 5-Fluorouracil, Gemcitabine, and Gefitinib. Moreover, paclitaxel and docetaxel are both anti-cancer agents belonging to the taxane family and can inhibit cancer cell proliferation by inducing cell cycle arrest (Ashrafizadeh et al., 2021). On the basis of the GSEA above, cell cycle-related pathways, such as cell cycle, spliceosome, and base excision repair, were apparently activated with increased risk scores, which shed light on the higher sensitivity of high-risk patients to Paclitaxel and Docetaxel. To summarize, considering the strong correlation of the prognostic signature with both immunotherapy and chemotherapy, we present a valid and robust tool that can guide clinicians to make effective personalized treatment decisions for patients with OSCC with different risk levels.

Despite considerable strides in treatment regimens and new drugs for OSCC, the survival rate has been poor and unsatisfactory in recent decades (Bray et al., 2018; Nör and Gutkind, 2018). Thus, the development of novel drugs for OSCC is still necessary. The new use of old drugs is more cost-effective compared to the exploration and development of novel drugs (Cheng et al., 2021). CMap, a public database for predicting small molecule drugs according to transcriptional expression data, has been used to predict and screen potential novel drug candidates for cancers in previous studies (Cheng et al., 2021; Wang M. et al., 2021). Therefore, to uncover novel drug candidates for patients with OSCC with high-risk scores, we evaluated DEGs between the two risk subgroups and finally screened out 10 potential therapeutic agents, including Dextromethorphan and AH-6809, in the CMap database, all of which have been barely explored in OSCC. Dextromethorphan is a safe FDA-approved drug with few undesirable side effects and usually serves as an effective antitussive agent (Silva and Dinis-Oliveira, 2020). Wang et al. found that Dextromethorphan and Metformin, at their pharmacological doses, could synergistically repress nicotine-enhanced cancer-initiating cell properties and halt tumor progression by directly targeting CHRNA7 to inhibit JAK2/STAT3/SOX2 signaling in esophageal squamous cell carcinoma and perhaps other nicotine-sensitive cancer types (Wang et al., 2021a). Additionally, AH-6809 was reported to inhibit the proliferation of non-small cell lung cancer cells by antagonizing prostaglandin receptors (Casibang and Moody, 2002). Hence, the results of CMap analysis may provide some new promising drug candidates for high-risk patients with OSCC and identify a viable direction for the future research on chemotherapy drugs.

## Conclusion

In summary, we established a novel prognostic signature with six OSGs. On the basis of both TCGA-OSCC and GSE41613 cohorts, the signature was proven to be an independent prognostic factor with high accuracy, as well as an impactful indicator for predicting the prognosis and immune status of patients with OSCC. Meanwhile, the constructed signature demonstrated that patients with high-risk scores might benefit more from ICI treatment compared to those with low-risk scores, and the risk score presented a close interaction with the TME and chemotherapy sensitivity. Our findings may also provide valuable new insight into the roles of oxidative stress in the prognosis, TME, immune status, immunotherapy response, and chemotherapy sensitivity of patients with OSCC. The oxidative stress-related signature may provide a valid and robust tool that can not only efficiently predict the prognosis and immune status but also guide clinicians to develop effective personalized therapeutic strategies for patients with OSCC.

## Data Availability

All datasets presented in this study are included in the article/Supplementary Material.

## Supplementary material

Supplementary Figure S1 A forest plot of the univariate Cox regression analysis with the prognostic ORGs **(A)** and the 1-, 3- and 5-year ROC curves and AUC values of the signature in TCGA cohort **(B)** and GEO cohort **(C)**. OSGs, Oxidative stress-related genes, ROC, Receiver operating characteristic, AUC, Area under curve, TCGA, The Cancer Genome Atlas, GEO, The Gene Expression Omnibus.

Supplementary Figure S2 Expression levels of model genes between low- and high-risk subgroups in TCGA **(A)** and GEO **(B)** cohorts. *** *p* < 0.001. GEO, The Gene Expression Omnibus, TCGA, The Cancer Genome Atlas.

Supplementary Figure S3 Survival analyses of model genes in OSCC using Kaplan-Meier algorithm based on TCGA **(A-F)** and GEO **(G-L)** cohorts. OSCC, Oral squamous cell carcinoma, GEO, The Gene Expression Omnibus, TCGA, The Cancer Genome Atlas.

Supplementary Figure S4 Survival analyses of clinical features in OSCC using Kaplan-Meier algorithm based on TCGA and GEO cohorts. TCGA cohort: **(A)** age, **(B)** gender, (C) grade, **(D)** clinical stage, **(E)** T stage and **(F)** N stage. GEO cohort: **(G)** age, **(H)** gender, and **(I)** clinical stage. OSCC, Oral squamous cell carcinoma, GEO, The Gene Expression Omnibus, TCGA, The Cancer Genome Atlas.

Supplementary Figure S5 Correlations between the risk score and the levels of immune score, stromal score and tumor purity according to TCGA **(A-C)** and GEO **(D-E)** cohorts. GEO, The Gene Expression Omnibus, TCGA, The Cancer Genome Atlas.

Supplementary Figure S6 Immune cells and immune-related functions with prognostic values in OSCC on basis of TCGA and GEO cohorts. OSCC, Oral squamous cell carcinoma, GEO, The Gene Expression Omnibus, TCGA, The Cancer Genome Atlas.

Supplementary Figure S7 The IC50 of selected chemotherapeutic agents between different risk subgroups of OSCC and correlations between the risk score and the IC50 of selected chemotherapeutic agents in TCGA cohort based on CGP, GDSC2 and CTRP databases. OSCC, Oral squamous cell carcinoma, IC50, Half-maximal inhibitory concentration, TCGA, The Cancer Genome Atlas, CGP, the Cancer Genome Project, GDSC, Genomics of Drug Sensitivity in Cancer, CTRP, Cancer Therapeutics Response Portal.

Supplementary Figure S8 The 2D chemical structures of the selected small molecule candidates for treating OSCC patients using CMap algorithm online. OSCC, Oral squamous cell carcinoma, CMap, the Connectivity Map.
