# Supplementary figures and images for: An oxidative stress-related prognostic signature for indicating the immune status of oral squamous cell carcinoma and guiding clinical treatment
Source: Front Genet. 2022 Sep 23;13:977902. doi: 10.3389/fgene.2022.977902 (PMC9538189; doi:10.3389/fgene.2022.977902)

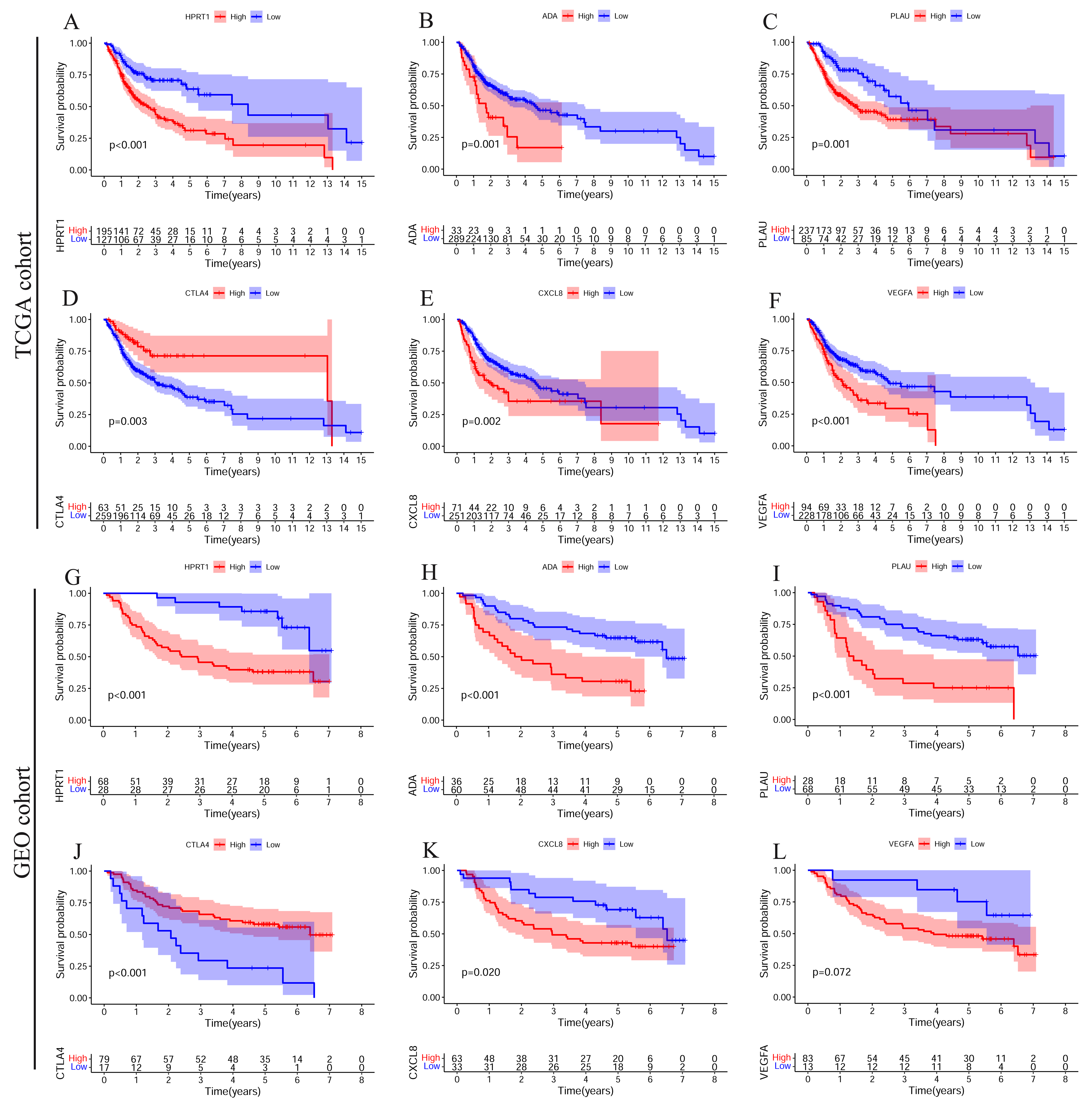

Supplement: Supplementary file 1 [file Image3.JPEG]

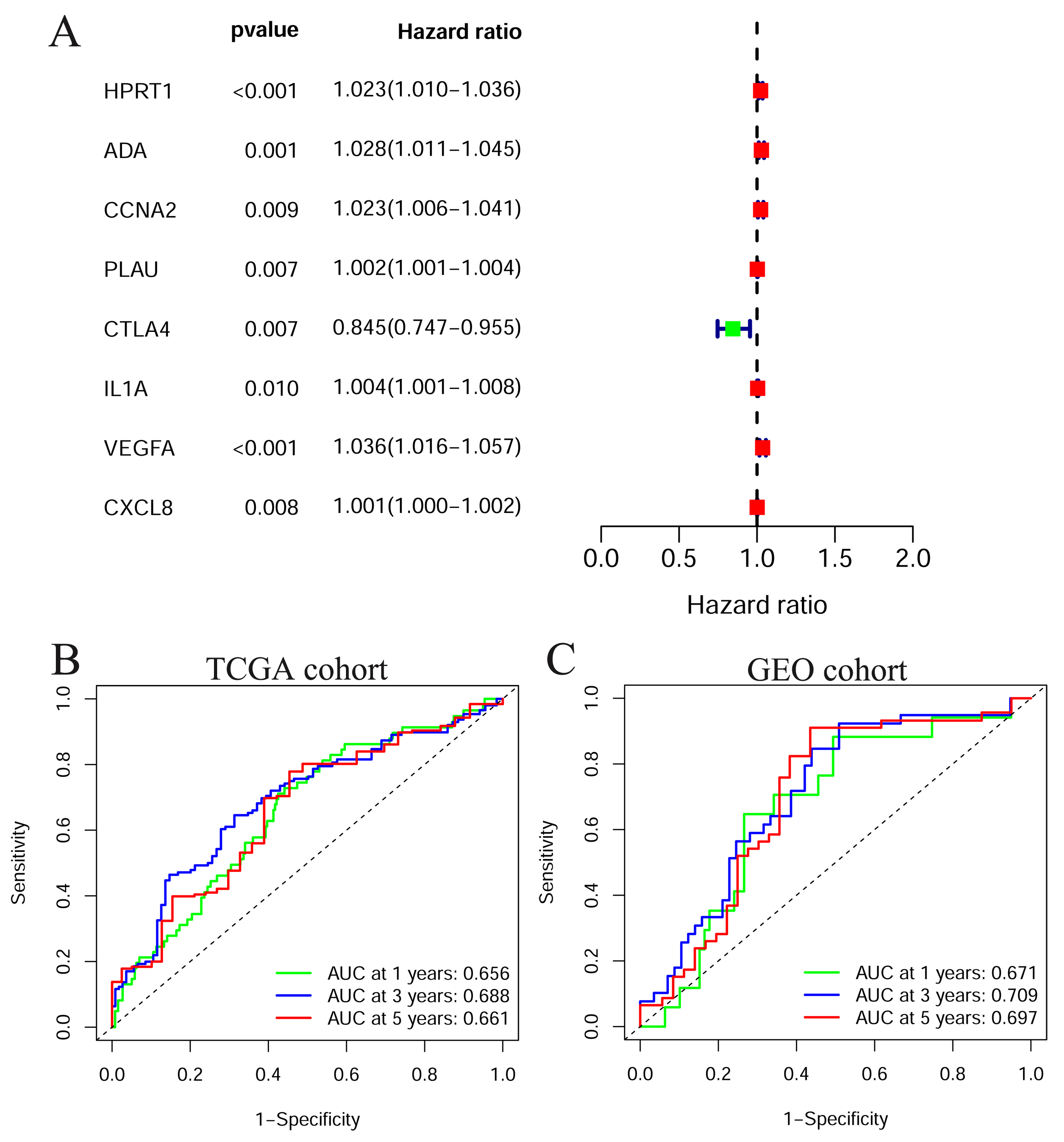

Supplement: Supplementary file 3 [file Image1.JPEG]

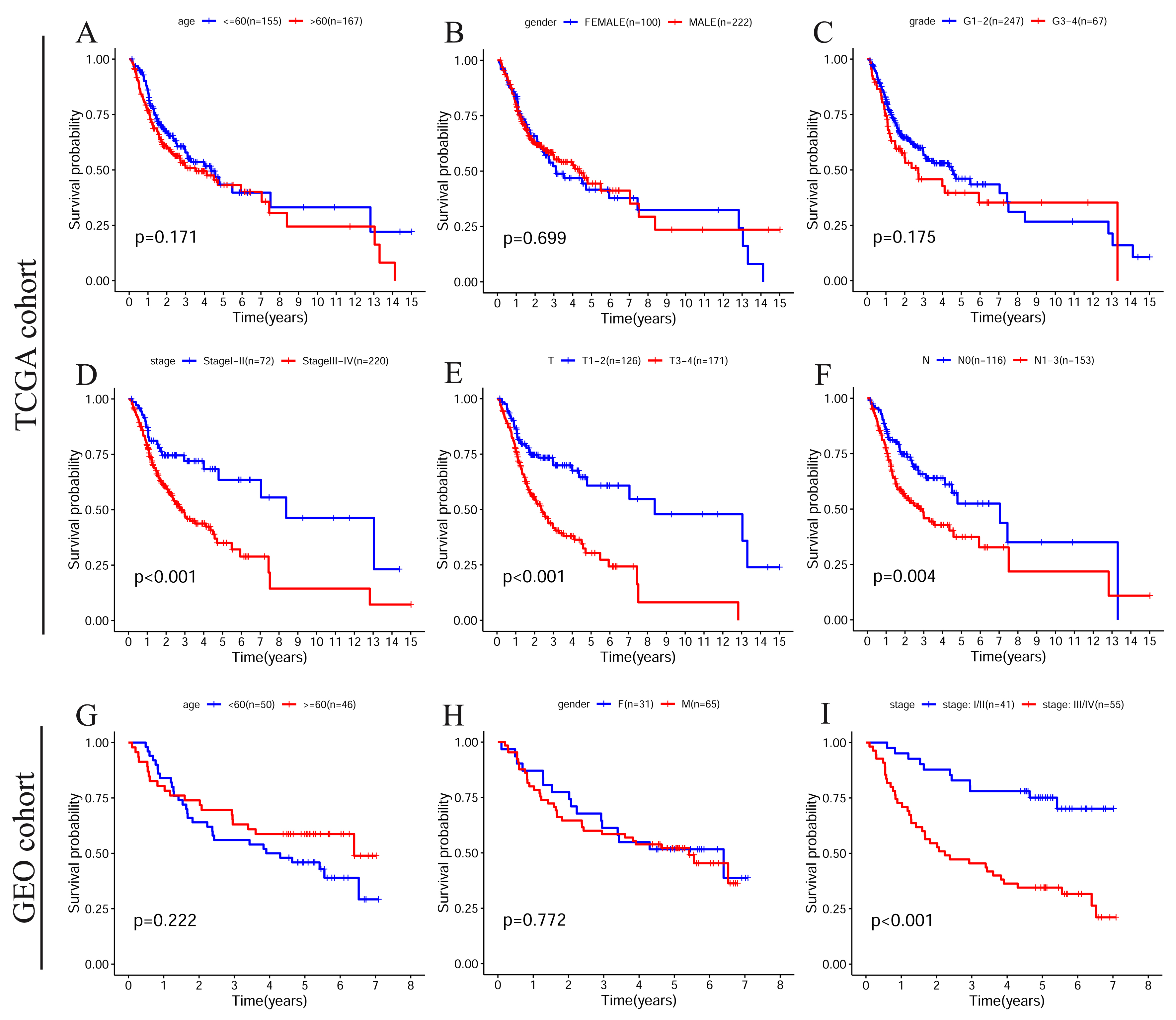

Supplement: Supplementary file 4 [file Image4.JPEG]

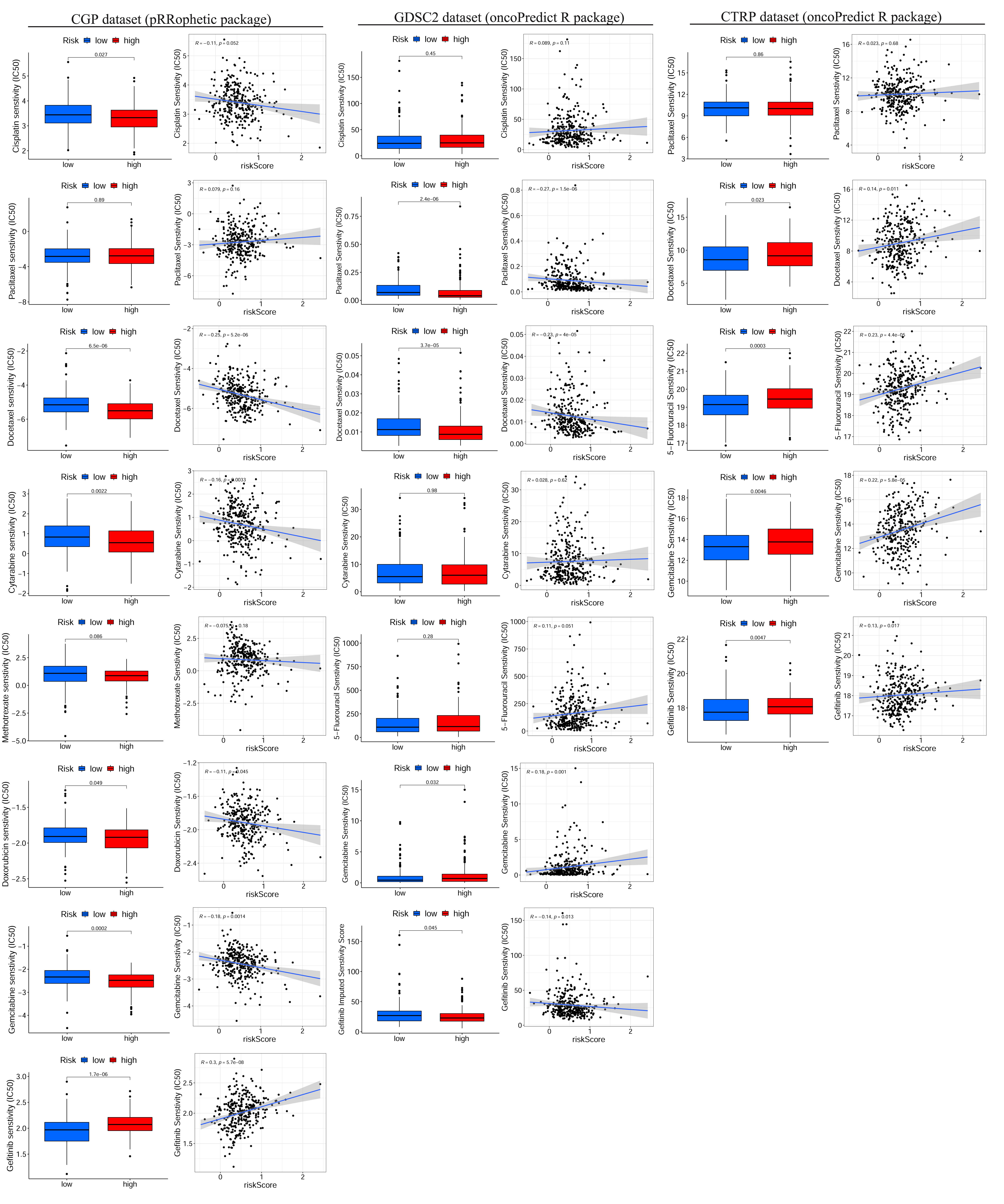

Supplement: Supplementary file 5 [file Image7.JPEG]

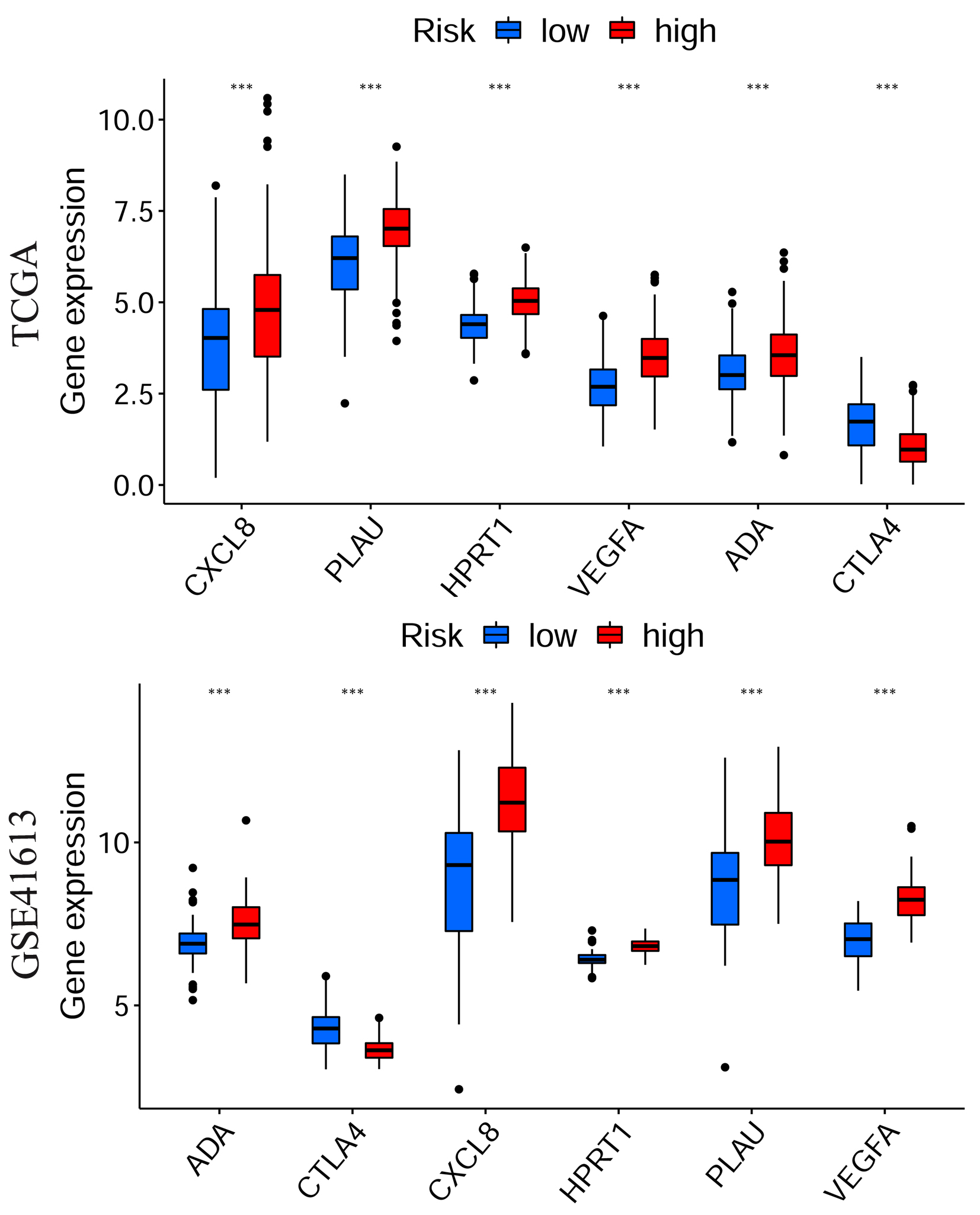

Supplement: Supplementary file 6 [file Image2.JPEG]

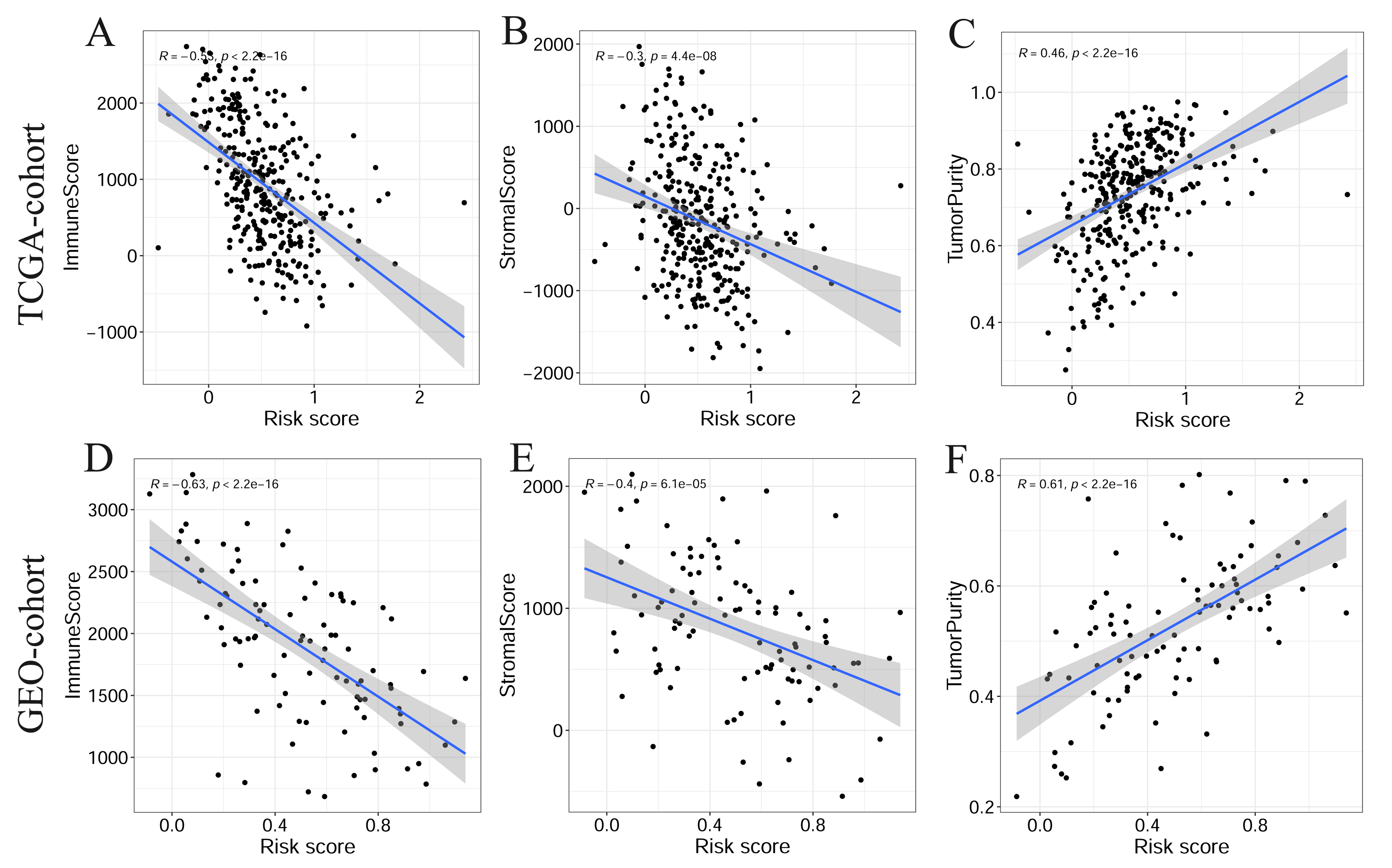

Supplement: Supplementary file 7 [file Image5.JPEG]

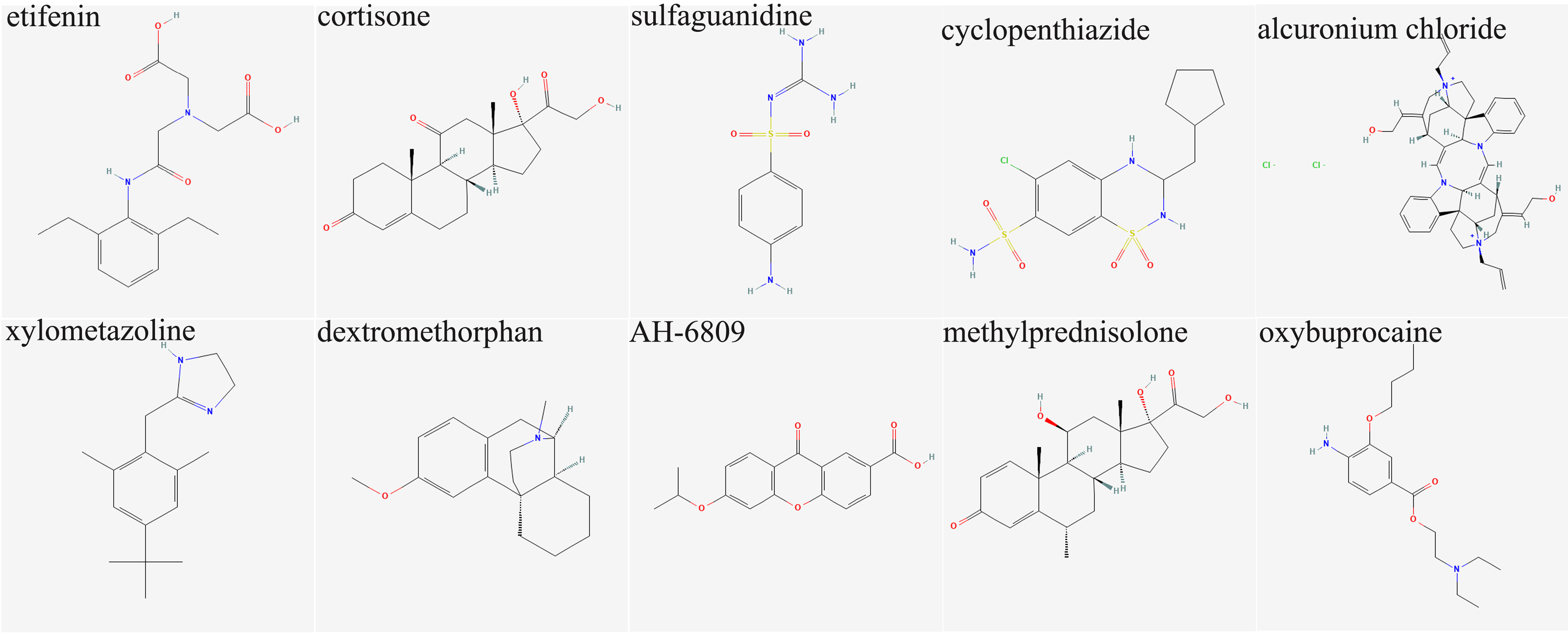

Supplement: Supplementary file 11 [file Image8.JPEG]

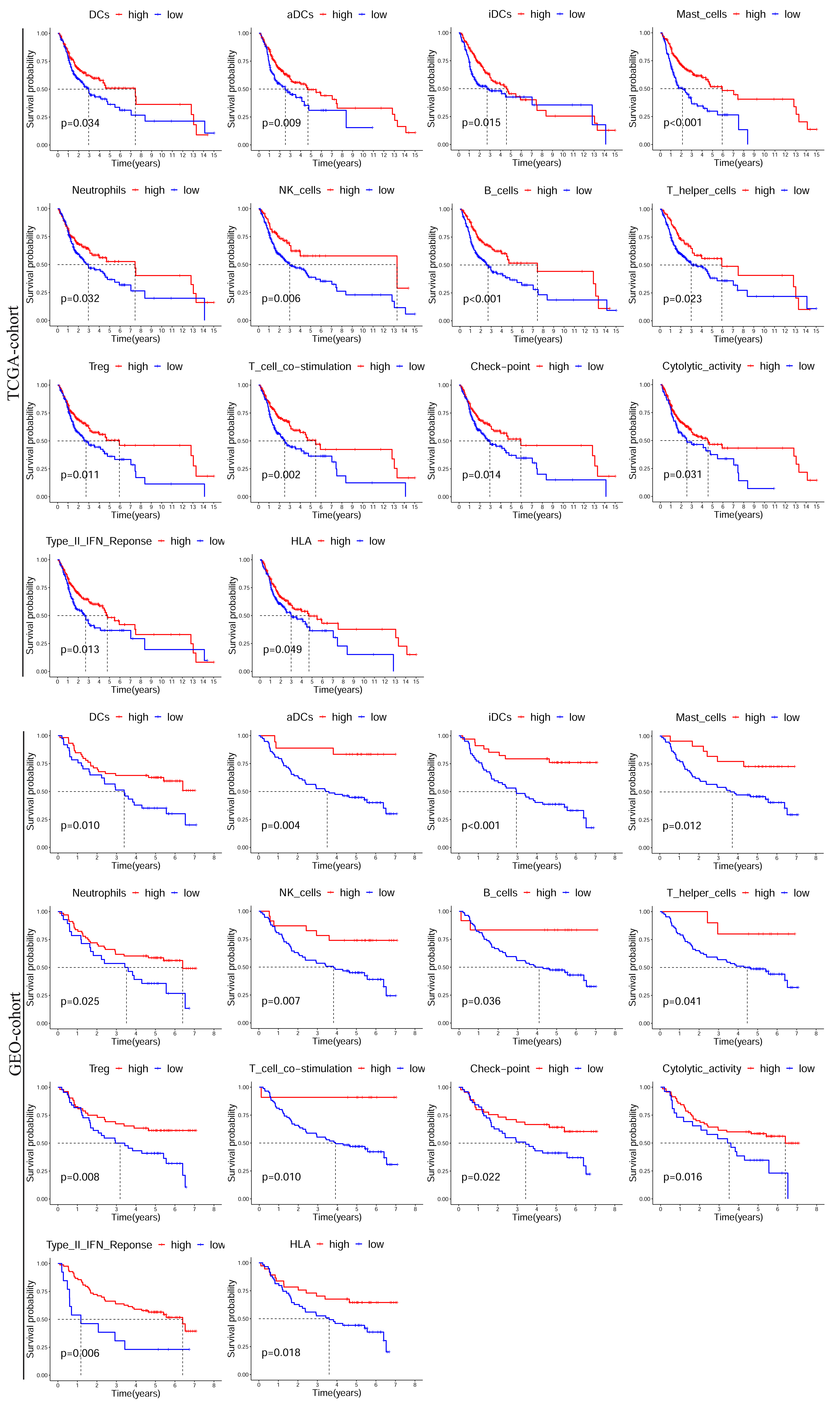

Supplement: Supplementary file 12 [file Image6.JPEG]
