# Supplementary material for: An oxidative stress-related prognostic signature for indicating the immune status of oral squamous cell carcinoma and guiding clinical treatment
Source: Front Genet. 2022 Sep 23;13:977902. doi: 10.3389/fgene.2022.977902 (PMC9538189; doi:10.3389/fgene.2022.977902)
Supplement: Supplementary file 2 [file Table1.DOCX]

**Supplementary Table S1.** Clinical characteristics of OSCC patients from TCGA and GEO datasets in the study.

| **Variable** | **No. of samples in TCGA** | **No. of samples in GEO** |
| --- | --- | --- |
| Gender |  |  |
| Male/Female | 222/100 | 65/31 |
| Age at diagnosis |  |  |
| <=60/>60 | 155/167 | 50/46 |
| Tumor grade |  |  |
| G1-2/G3-4 /unknown | 247/67/8 | NA |
| Clinical stage |  |  |
| I-II/III-IV/unknown | 72/220/30 | 41/55/0 |
| T stage |  |  |
| T1-2/T3-4/unknown | 126/171/25 | NA |
| M stage |  |  |
| M0/M1/unknown | 116/0/206 | NA |
| N stage |  |  |
| N0/N1-3/unknown | 116/153/53 | NA |

OSCC, oral squamous cell carcinoma; TCGA, the Cancer Genome Atlas;

GEO, Gene Expression Omnibus; NA, Not Available.
