# Supplementary material for: An oxidative stress-related prognostic signature for indicating the immune status of oral squamous cell carcinoma and guiding clinical treatment
Source: Front Genet. 2022 Sep 23;13:977902. doi: 10.3389/fgene.2022.977902 (PMC9538189; doi:10.3389/fgene.2022.977902)
Supplement: Supplementary file 8 [file Table2.DOCX]

**Supplementary Table S2.** Oxidative stress-related genes present in both TCGA-OSCC and GSE41613 cohorts.

| KIT | TTPA | ERCC8 | TLR3 | PRKG1 | GTPBP3 |
| --- | --- | --- | --- | --- | --- |
| NDUFA13 | RARA | DDAH1 | BAD | TGFB3 | RHOA |
| MAPK13 | UCN | NDUFS7 | TBP | BACE1 | PPARA |
| TNFSF4 | PKP2 | NDUFS6 | TJP1 | SNTA1 | ABL1 |
| OXTR | KCNT1 | DSP | ALDH9A1 | DRD5 | KDR |
| DHFR | MGMT | NDUFA10 | BIRC5 | SERPINA3 | SPP1 |
| ACACA | SMPD1 | NRAS | FCGR3B | HSPA9 | ACAD9 |
| GPX8 | DMPK | IGF2BP1 | PDGFRL | AMPD1 | AGT |
| TAZ | PYCR1 | CXCL9 | NLRP1 | TNFRSF1B | TRPM2 |
| YBX1 | UTRN | BCL2L11 | GAD1 | ITPR1 | PIK3CA |
| SFXN4 | FOXJ1 | STK4 | ENO2 | TSFM | HMGB1 |
| HPSE | CCR5 | HNF4A | PEX12 | UCP3 | CLU |
| RPTOR | SLC40A1 | ALOX12 | CDKN1B | DNM1L | CDKN3 |
| MTA1 | CCL11 | GLS2 | GJA1 | MET | HBB |
| CD274 | MECOM | CFH | DRD4 | PGD | NDUFA12 |
| ENDOG | DUSP19 | F8 | IRF1 | HMOX2 | SELP |
| BMP4 | VASP | DNAJB1 | ATF2 | PPIF | SERPINE1 |
| MTTP | FH | TBK1 | CDK1 | EHHADH | C1QBP |
| TCF7L2 | NOSIP | ITIH4 | RNF112 | TXNRD2 | PRDX3 |
| TLR6 | ESR2 | XRCC1 | HMGCR | AKR1B1 | TXNRD1 |
| VDR | PIK3C3 | TP73 | DHCR24 | GSTM2 | MSRB1 |
| GADD45G | IL33 | ANGPT2 | AHSP | NGB | AGER |
| FMO4 | CXCR1 | AOX1 | NDUFA6 | MMP1 | PRL |
| AREG | PRKCG | NME1 | PLCG1 | NCF1 | LDLR |
| HK1 | DECR1 | DES | IRAK1 | MECP2 | TRDN |
| PIK3R2 | NR1H2 | TRPA1 | FMO1 | NPPB | CASQ2 |
| ATG5 | AKAP9 | TGFA | HSP90AB1 | SIRT2 | PC |
| SIRT6 | MSR1 | MAP3K7 | ITGAL | STAT1 | CALM1 |
| JAZF1 | RPA1 | EPX | AKT2 | ECHS1 | ETFB |
| FADD | RCAN1 | RHOD | CCR6 | AOC3 | SHC1 |
| BCL2A1 | FTH1 | GP1BA | KIF1B | H6PD | COMT |
| IL11 | CYP11A1 | LGALS3 | DSPP | FGF2 | ANXA5 |
| MAP3K11 | SENP3 | CYC1 | CCNA2 | GLRX2 | MMP2 |
| ASPA | RRM2B | BAG3 | CPOX | KRIT1 | TH |
| ROCK1 | PDK1 | STAT4 | ALDH1A1 | SDHAF1 | SELE |
| NEDD8 | SNCB | NR1H4 | HSPB2 | SESN2 | STAT3 |
| GAA | CAPN3 | FCGR2B | MT3 | REN | NUDT1 |
| PEPD | RNASE3 | CSK | SDHAF2 | ADRB2 | EIF2S1 |
| CAPN2 | PRPH | STK24 | EPAS1 | NR3C2 | EIF2AK3 |
| PTPRC | CFI | AR | LONP1 | GSK3B | TNFRSF1A |
| CYP20A1 | LAMP1 | GYG1 | ABCC1 | KRAS | TERT |
| MKI67 | BLOC1S1 | PRKD2 | SETD2 | ADH5 | GDNF |
| SORL1 | PML | PPIA | E2F1 | FXN | SLC2A1 |
| SORD | TIMP2 | CDK4 | MTFMT | PTK2 | SOD3 |
| MUC5AC | TRAF2 | IRF5 | CALB1 | HLA-DRA | MB |
| ALDH3A2 | STIP1 | F5 | MBP | UGT1A1 | CYP1A2 |
| CHCHD2 | NOSTRIN | SCO2 | S100A9 | PRKD1 | NR3C1 |
| PLD1 | MYLK | PKM | NDUFS1 | CYB5R3 | MMP9 |
| TNFRSF11B | GSTM5 | GLO1 | IGF1R | GPT | TXN2 |
| TNFRSF10A | TPK1 | MYH6 | CHUK | F3 | OXSR1 |
| PFKM | SLC2A4 | MME | DNASE1 | TFRC | C9orf72 |
| IL6R | HLA-A | FOXM1 | IFNB1 | IL1R1 | IGF1 |
| SUMO1 | UNG | ACOX2 | VIM | MAP2K1 | TPO |
| TEK | IL3 | JAK1 | ANK2 | ATP2A2 | EGF |
| SLC7A1 | RORA | NDRG1 | MAP2K7 | PLA2G7 | MYH7 |
| FIG4 | MDH1 | PEX11B | CR2 | CD40 | MSRA |
| GLT8D1 | RUNX2 | ERBB2 | DLG4 | GSTO1 | CYP2E1 |
| LBR | SCARA3 | FKBP5 | DRD1 | BCL2L1 | GCH1 |
| CXCL16 | PDIA3 | IL2RA | PCNA | ACHE | ELAC2 |
| OSM | GSTM4 | MSH2 | ADCY10 | CRAT | MAOB |
| GRIN1 | PIGA | RAB5A | PLAT | GCLC | PRDX6 |
| DYRK1A | BRCA2 | PLAUR | TSC1 | NFKBIA | CYP1A1 |
| DNM2 | UNC13A | SPR | ELN | UCP2 | PINK1 |
| TACO1 | TNFSF11 | LCK | MBL2 | NR4A2 | GPX3 |
| GIGYF2 | CYP2C8 | MSRB3 | BMP2 | CD40LG | ACADL |
| C5AR1 | SERPINF1 | GLE1 | LCN2 | EGR1 | SP1 |
| ALDH3B1 | PSIP1 | TAF15 | GLUD1 | TRMT10C | NOX4 |
| CCR7 | TPI1 | GFM2 | TNFSF10 | PNPT1 | CASP9 |
| MUC1 | PDGFRB | SETX | DMD | CDKN2A | F2 |
| CANX | CAMK2G | GHRL | MCL1 | PTK2B | ETFA |
| ADH1A | EPHX2 | KCNMA1 | NOL3 | CCL5 | PPARG |
| FRZB | CR1 | FOXP3 | CST3 | NPM1 | HTRA2 |
| EEF2 | CYP3A5 | KRT8 | CBS | ETS1 | ADIPOQ |
| ZFAND1 | AGRN | PLA2G2A | DDAH2 | ACO1 | CYP3A4 |
| UBQLN4 | XRCC6 | BRF2 | NTF3 | EEF1A1 | ALDH2 |
| TNIP1 | PPP3CA | FKRP | ACTB | TAC1 | COX5A |
| SCN4B | UBQLN1 | ALAD | PRKAB1 | CASP1 | OGG1 |
| ARNT | FOXO4 | NDUFB9 | STUB1 | PRKCD | MTOR |
| SDC1 | MAPK7 | ACTN2 | FCGR2A | SLC5A7 | ABCD1 |
| CACNA2D1 | FTL | ISCU | ITGB2 | SMAD3 | MTO1 |
| AQP1 | BACH1 | XIAP | MATR3 | ENG | TLR4 |
| CDC25C | SCGB1A1 | CFLAR | APOB | GLUL | BAX |
| ACTN4 | FMO2 | NLRP3 | STK25 | HSD17B4 | PRDX2 |
| BSG | APC | QDPR | TLR9 | RAC2 | POLG |
| LYRM4 | MYD88 | CASP2 | CSF2 | PON3 | MAPK3 |
| KL | HERPUD1 | TYMP | APAF1 | ATXN2 | VCAM1 |
| ANXA11 | CYP17A1 | SCO1 | TNFAIP3 | PTEN | HSF1 |
| ABCC3 | TYRP1 | PTS | ABCB1 | TSPO | NCF2 |
| MRAP | XRCC5 | SYP | TGM2 | ELANE | PRNP |
| FANCD2 | FKBP1B | TYK2 | ACTA1 | SDHC | FOS |
| FIS1 | MMP14 | GRN | GRB2 | TUFM | MAOA |
| PGAM5 | TMEM161A | OPTN | MRPS14 | ERN1 | APOE |
| CAMKK2 | ALOX15 | GRIA1 | LTF | DBH | HIF1A |
| VIPR1 | ASL | PIK3CB | CCL4 | CYP2C9 | CACNA1C |
| ADRB3 | CCNF | IKBKB | NDUFS2 | GPX7 | CASP8 |
| RTN4 | DIABLO | CEBPB | BTD | P4HB | LMNA |
| FAAH | TRPV4 | CD80 | IFNA1 | FUS | PPARGC1A |
| GLA | PGK1 | PPP1R15A | RB1 | FASLG | CAV1 |
| PDE4A | NDUFA1 | ACSL4 | SMAD4 | TF | PON2 |
| CX3CR1 | IL23A | TUBA1B | MAP2K6 | CHKB | BCL2 |
| NOD2 | ATXN1 | STK11 | SRXN1 | CDK2 | TARDBP |
| SFTPB | JUNB | MMP3 | LPO | CD36 | ACE |
| LANCL1 | HSPA14 | SMARCA4 | IRS1 | HFE | APEX1 |
| CD38 | CD86 | NTRK1 | SLC17A5 | ACP1 | OLR1 |
| FECH | FGF7 | ECE1 | HTR2C | GSTA1 | ESR1 |
| TPPP3 | SCP2 | LYN | ENO1 | NOTCH1 | MAP3K5 |
| IL1RAPL2 | BCL6 | ELK1 | LAMP2 | DUSP1 | VCP |
| TP53INP1 | GSN | ALDH3A1 | ITGB1 | PRDX4 | AIFM1 |
| ENC1 | MDH2 | CCS | SERPINA1 | PTPN11 | ICAM1 |
| PEX5 | IL2RB | SIL1 | S100B | AGTR1 | CP |
| IKBKG | ABCC8 | PSEN2 | REST | HSP90B1 | BDNF |
| FYN | PPIG | ARG2 | PIK3R1 | FN1 | NFKB1 |
| ABCG2 | MCU | IDO1 | CDC42 | PRKCB | PRDX5 |
| UCN2 | MLYCD | MTR | CXCL12 | CNR1 | CAV3 |
| CD46 | CHMP2B | PDIA2 | DAO | TREM2 | TYR |
| KIAA0319L | UBC | SLPI | BAK1 | NDUFV2 | KEAP1 |
| CXCL2 | MSN | SLC1A1 | MTHFR | ITGAM | GSTM1 |
| CD28 | PTPN3 | EIF2B4 | CSF3 | TECRL | AARS2 |
| GADD45B | SLC19A3 | ADAM17 | BRCA1 | IL1RN | FOXO1 |
| EIF2AK4 | CCNB1 | CYP2A6 | GNAS | CACNA1S | PSEN1 |
| NEK1 | FMR1 | HDAC1 | SIGMAR1 | HTT | DDIT3 |
| HDAC9 | HSPA6 | CD79A | IREB2 | PLAU | GSTP1 |
| ACAD8 | HK2 | CYP27A1 | TGFBR1 | TIMP1 | CPT1A |
| ADAM10 | SELL | RXRA | CARS2 | CDK5 | SLC25A20 |
| RAG2 | PRKCZ | PTPN1 | MAP2 | CALR | SQSTM1 |
| C3 | PTGIS | EP300 | MIF | MGST1 | ETFDH |
| HRH2 | AHR | CXCR3 | CTSD | SUOX | HSPA5 |
| VEGFC | GLS | NAT2 | MAPKAPK3 | PTGS1 | ACOX1 |
| SLC8A1 | PPOX | EDNRA | OXR1 | CYP2C19 | CYP2D6 |
| MRPS34 | NCAM1 | PFN1 | CACNB4 | TTN | NDUFS4 |
| IL12B | RETN | NTHL1 | PXN | DLD | GAPDH |
| CIITA | B2M | KCNJ2 | HTR3A | CS | MAPT |
| SIAH1 | CD55 | NRG1 | IGF2R | TXNIP | PARP1 |
| SET | VKORC1L1 | CHCHD10 | CASP7 | IL18 | VEGFA |
| MMD | SLC25A27 | BECN1 | GSTO2 | GCDH | RYR2 |
| ITGA2 | EDNRB | CLEC4A | ANG | CYGB | CCL2 |
| TLR5 | PF4 | COQ2 | EPHA3 | CCND1 | HADHB |
| PHYH | SMAD2 | EIF4EBP1 | PTPN22 | PDHA1 | RYR1 |
| HSPG2 | SOCS1 | ANGPT1 | MRPS16 | LRRK2 | CYBB |
| PRDM10 | PYGM | SOCS3 | GLUD2 | HTR2A | GFM1 |
| RBP4 | CDK6 | AIF1 | CREBBP | DRD2 | HSPA4 |
| DYNC1H1 | MICB | IL15 | SLC25A13 | SLC6A3 | PTGS2 |
| HPRT1 | PPARD | TRIM21 | GSTA4 | MAPKAPK2 | SNCA |
| SPARC | UBQLN2 | LTA | IDH2 | ARG1 | IFNG |
| MAPK8IP1 | HYOU1 | NAMPT | TOR1A | GGT1 | NQO1 |
| TFEB | TACR1 | MFN2 | DCTN1 | SLC25A4 | HSP90AA1 |
| HDAC2 | BDKRB2 | TIA1 | C12orf65 | NPY | EDN1 |
| VNN1 | ADRB1 | RELA | EPHX1 | BMP6 | TGFB1 |
| SORCS2 | CTTN | HGF | BCHE | HSPD1 | JUN |
| ADSL | DEPDC5 | GAP43 | ERCC6 | CYP1B1 | CYBA |
| NEFL | NQO2 | SLC6A2 | NFE2L1 | NOX1 | CXCL8 |
| OTC | ABCC2 | CD4 | EIF2AK2 | HP | SIRT1 |
| LCAT | SREBF1 | PENK | ADM | BLVRB | ACADS |
| RYR3 | SERPINH1 | CFTR | COL2A1 | NOS1AP | HADH |
| ISG15 | VCL | RPS6KA5 | VIP | FARS2 | AKT1 |
| MAP3K1 | CASP4 | CTSB | SYK | HRAS | IL10 |
| BCR | CD34 | ERBB4 | A2M | RAC1 | ACADVL |
| SLC25A1 | KRT18 | EIF4G1 | CD44 | SNAP25 | TXN |
| EPHA4 | CTSG | KLF4 | IAPP | PRKAA2 | CASP3 |
| MAPKAPK5 | SLC11A2 | CYP2B6 | PRKCA | PRKAA1 | G6PD |
| CLIC1 | ODC1 | ACE2 | SNCAIP | EPO | MAPK1 |
| SLC7A11 | TNFRSF10B | SESN1 | S100A8 | MSRB2 | SLC6A4 |
| TLR8 | CHEK1 | NRF1 | NAGS | LOX | ACADM |
| NCF4 | CD69 | PECAM1 | HNRNPA1 | NDUFB8 | IL1B |
| DGKQ | PDLIM4 | KLF2 | ATP13A2 | PDE5A | CYCS |
| IFNAR1 | GH1 | GSS | SLC25A3 | CRYAB | MAPK8 |
| MMP8 | LPL | EIF2AK1 | DAPK1 | EGFR | HADHA |
| CDKN2B | HNF1A | FLT1 | TTR | MDM2 | IL6 |
| TOP1 | SCARB1 | RPS6KB1 | DLST | THBD | PON1 |
| ACO2 | PTX3 | OXA1L | UBE2L3 | NDUFV1 | PARK7 |
| CUL3 | RAD51 | ATXN3 | G3BP1 | CTLA4 | GSR |
| DUOX1 | EZH2 | HLA-B | ADH1C | MAPK9 | XDH |
| CAMK4 | LGALS1 | ATF3 | DNMT1 | NGF | APP |
| CUL1 | VAPB | RAF1 | MRPS22 | IL2 | MAPK14 |
| CYB5A | ELAVL1 | MALAT1 | TFAM | SRC | MPO |
| IGF2BP2 | HRH1 | ADA | OSGIN2 | CREB1 | SOD2 |
| UBE2D2 | ATR | MAPK11 | SCN4A | ATF6 | CPT2 |
| FAM120A | PNKP | GRIN2B | UCHL1 | TLR2 | NFE2L2 |
| AQP4 | GFER | ANXA2 | FGFR1 | ATM | NOS1 |
| PDGFB | ABCA1 | COX6B1 | OXT | SDHD | TNF |
| BACH2 | DAXX | HMGCL | AKR1A1 | IL1A | CAT |
| PPP5C | CDH2 | CXCR4 | OPA1 | NDUFS3 | SOD1 |
| AURKA | GZMB | CXCL10 | SLC1A2 | CDKN1A | NOS3 |
| TLR7 | CALB2 | MMP13 | CRHR1 | SLC1A3 |  |
| WRN | MUTYH | STK39 | POR | ATF4 |  |

TCGA, the Cancer Genome Atlas; OSCC, oral squamous cell carcinoma.
