# Supplementary material for: An oxidative stress-related prognostic signature for indicating the immune status of oral squamous cell carcinoma and guiding clinical treatment
Source: Front Genet. 2022 Sep 23;13:977902. doi: 10.3389/fgene.2022.977902 (PMC9538189; doi:10.3389/fgene.2022.977902)
Supplement: Supplementary file 9 [file Table3.DOCX]

**Supplementary Table S3.** Differentially expressed ORGs in OSCC tissues vs. adjacent normal tissues.

| **Gene** | **N-Mean** | **T-Mean** | **logFC** | **pValue** | **FDR** |
| --- | --- | --- | --- | --- | --- |
| KIT | 5.85547 | 1.989583 | -1.55732 | 8.15E-08 | 3.40E-07 |
| TNFSF4 | 0.881464 | 1.821149 | 1.046875 | 6.50E-13 | 1.04E-11 |
| GPX8 | 3.161454 | 7.24977 | 1.197347 | 1.10E-11 | 1.19E-10 |
| CD274 | 2.186594 | 4.619337 | 1.079 | 0.001094 | 0.001993 |
| GADD45G | 10.57357 | 2.87899 | -1.87683 | 0.000729 | 0.001392 |
| FADD | 3.669011 | 15.18978 | 2.049638 | 8.19E-16 | 4.74E-14 |
| IL11 | 0.603629 | 3.552114 | 2.556944 | 4.30E-19 | 4.77E-16 |
| ASPA | 1.036276 | 0.5094 | -1.02454 | 4.58E-16 | 2.98E-14 |
| MKI67 | 3.907057 | 12.24509 | 1.648049 | 8.10E-13 | 1.20E-11 |
| OSM | 1.46727 | 3.216301 | 1.132268 | 0.000231 | 0.000485 |
| FRZB | 7.256253 | 1.421999 | -2.3513 | 4.40E-11 | 4.00E-10 |
| AQP1 | 41.51417 | 17.41658 | -1.25314 | 6.40E-06 | 1.82E-05 |
| CDC25C | 0.838818 | 2.202507 | 1.392717 | 3.73E-17 | 4.59E-15 |
| UCN2 | 1.353964 | 4.603563 | 1.765562 | 3.26E-14 | 9.17E-13 |
| CXCL2 | 10.48996 | 4.515711 | -1.21598 | 0.019471 | 0.027659 |
| GADD45B | 39.97989 | 12.64445 | -1.66077 | 9.60E-06 | 2.65E-05 |
| VEGFC | 2.29112 | 11.09048 | 2.275196 | 3.02E-10 | 2.24E-09 |
| PHYH | 13.73791 | 4.041928 | -1.76505 | 5.15E-06 | 1.48E-05 |
| RBP4 | 2.645697 | 1.079143 | -1.29376 | 9.68E-08 | 3.97E-07 |
| HPRT1 | 10.20706 | 23.53007 | 1.204938 | 6.83E-15 | 2.61E-13 |
| SPARC | 41.09908 | 246.7903 | 2.586108 | 1.20E-11 | 1.30E-10 |
| SORCS2 | 1.865346 | 4.381896 | 1.232112 | 2.45E-06 | 7.51E-06 |
| NEFL | 2.330001 | 12.07234 | 2.373302 | 1.74E-08 | 8.45E-08 |
| ISG15 | 5.57501 | 313.077 | 5.811399 | 4.78E-15 | 1.96E-13 |
| SLC7A11 | 2.08418 | 5.880192 | 1.496383 | 0.000753 | 0.00143 |
| IGF2BP2 | 1.889716 | 14.95638 | 2.98452 | 6.04E-16 | 3.72E-14 |
| AQP4 | 1.433351 | 0.555674 | -1.36708 | 5.69E-12 | 6.92E-11 |
| AURKA | 2.383742 | 10.39312 | 2.124329 | 2.49E-18 | 9.20E-16 |
| FOXJ1 | 5.840147 | 1.17788 | -2.30981 | 0.006429 | 0.010148 |
| CCL11 | 1.593228 | 3.604701 | 1.177927 | 7.96E-14 | 1.73E-12 |
| IL33 | 13.40372 | 4.972452 | -1.4306 | 4.06E-10 | 2.88E-09 |
| CAPN3 | 3.63941 | 1.316565 | -1.46693 | 0.000839 | 0.001573 |
| PML | 6.063712 | 13.89535 | 1.196329 | 1.51E-09 | 9.52E-09 |
| TRAF2 | 4.461217 | 9.05591 | 1.021422 | 1.98E-07 | 7.63E-07 |
| NOSTRIN | 2.467909 | 1.013046 | -1.28459 | 5.35E-07 | 1.87E-06 |
| SLC2A4 | 7.957613 | 0.85897 | -3.21166 | 1.86E-12 | 2.58E-11 |
| HLA-A | 188.2182 | 512.2923 | 1.444561 | 3.56E-09 | 2.00E-08 |
| SCARA3 | 13.55558 | 5.390698 | -1.33034 | 2.54E-08 | 1.19E-07 |
| PDIA3 | 48.41406 | 96.95125 | 1.001833 | 1.32E-13 | 2.49E-12 |
| PDGFRB | 5.96936 | 15.89291 | 1.412735 | 1.75E-07 | 6.76E-07 |
| EPHX2 | 8.18213 | 3.18251 | -1.36231 | 3.39E-14 | 9.17E-13 |
| CYP3A5 | 3.810213 | 1.150943 | -1.72706 | 6.84E-06 | 1.93E-05 |
| AGRN | 9.026334 | 43.16168 | 2.257539 | 8.56E-16 | 4.74E-14 |
| FTL | 727.8602 | 1558.751 | 1.098657 | 9.44E-08 | 3.89E-07 |
| SCGB1A1 | 25.96208 | 2.707665 | -3.26128 | 6.68E-05 | 0.000154 |
| FMO2 | 18.19561 | 2.672458 | -2.76735 | 9.46E-17 | 7.49E-15 |
| TYRP1 | 4.824287 | 0.712986 | -2.75837 | 0.000172 | 0.000369 |
| MMP14 | 26.3114 | 116.462 | 2.1461 | 1.76E-15 | 8.46E-14 |
| ALOX15 | 4.201407 | 1.180808 | -1.8311 | 0.000161 | 0.000349 |
| CCNF | 2.026848 | 5.323863 | 1.393236 | 1.28E-14 | 4.29E-13 |
| FGF7 | 2.507518 | 1.094301 | -1.19625 | 3.68E-09 | 2.06E-08 |
| MSN | 37.10242 | 116.341 | 1.648775 | 5.61E-14 | 1.24E-12 |
| CCNB1 | 7.978132 | 27.97092 | 1.809805 | 3.94E-14 | 1.04E-12 |
| GLS | 3.839455 | 7.966071 | 1.052967 | 7.30E-12 | 8.51E-11 |
| B2M | 320.7405 | 660.8681 | 1.042956 | 7.84E-08 | 3.29E-07 |
| SOCS1 | 2.478554 | 8.424075 | 1.76502 | 1.41E-12 | 2.00E-11 |
| PYGM | 113.5154 | 2.844167 | -5.31874 | 2.52E-11 | 2.54E-10 |
| CDK6 | 3.547872 | 9.467348 | 1.416006 | 2.60E-11 | 2.55E-10 |
| MICB | 1.887772 | 4.334474 | 1.199172 | 8.41E-13 | 1.21E-11 |
| CTTN | 21.46192 | 68.30257 | 1.670161 | 4.25E-07 | 1.51E-06 |
| SERPINH1 | 7.453729 | 61.66065 | 3.048316 | 1.39E-17 | 2.85E-15 |
| CTSG | 5.310243 | 2.005217 | -1.40502 | 0.000426 | 0.00085 |
| ODC1 | 20.62721 | 94.78405 | 2.200096 | 1.70E-05 | 4.41E-05 |
| TNFRSF10B | 6.254847 | 13.17547 | 1.074808 | 6.15E-10 | 4.18E-09 |
| CHEK1 | 1.769626 | 3.811954 | 1.107086 | 2.00E-15 | 9.22E-14 |
| LPL | 8.636693 | 2.572563 | -1.74727 | 0.000101 | 0.000225 |
| PTX3 | 7.689968 | 2.443249 | -1.65418 | 0.006557 | 0.01029 |
| RAD51 | 1.80494 | 5.143719 | 1.510861 | 9.18E-17 | 7.49E-15 |
| LGALS1 | 80.65402 | 320.2832 | 1.98953 | 1.78E-10 | 1.41E-09 |
| GZMB | 4.105676 | 9.238632 | 1.17006 | 0.000136 | 0.000298 |
| DDAH1 | 3.964908 | 1.869045 | -1.08499 | 2.14E-10 | 1.65E-09 |
| IGF2BP1 | 0.405864 | 1.364447 | 1.749249 | 6.27E-15 | 2.48E-13 |
| CXCL9 | 8.171062 | 45.06163 | 2.463304 | 1.91E-05 | 4.87E-05 |
| ALOX12 | 19.69958 | 4.42293 | -2.15509 | 0.000276 | 0.000574 |
| AOX1 | 3.545106 | 1.336012 | -1.4079 | 7.69E-13 | 1.17E-11 |
| DES | 743.4543 | 140.059 | -2.40821 | 8.52E-05 | 0.000192 |
| TGFA | 7.090195 | 15.28323 | 1.108053 | 2.06E-07 | 7.90E-07 |
| SCO2 | 12.46216 | 25.91078 | 1.055998 | 3.91E-07 | 1.41E-06 |
| PKM | 112.4057 | 240.6951 | 1.098491 | 5.61E-14 | 1.24E-12 |
| MYH6 | 4.827912 | 1.176722 | -2.03663 | 0.000312 | 0.000637 |
| FOXM1 | 2.590625 | 13.83816 | 2.41728 | 5.95E-17 | 6.27E-15 |
| ACOX2 | 2.18402 | 0.80149 | -1.44623 | 2.04E-10 | 1.59E-09 |
| NDRG1 | 55.71303 | 197.6442 | 1.826819 | 1.45E-09 | 9.16E-09 |
| PLAUR | 6.843276 | 14.77044 | 1.109954 | 2.89E-08 | 1.34E-07 |
| FOXP3 | 1.211417 | 3.159138 | 1.382835 | 3.72E-08 | 1.66E-07 |
| PLA2G2A | 40.83286 | 8.943567 | -2.19081 | 5.38E-14 | 1.24E-12 |
| ACTN2 | 87.93707 | 12.09616 | -2.86192 | 1.90E-05 | 4.87E-05 |
| TYMP | 35.61822 | 123.6489 | 1.795562 | 1.29E-10 | 1.04E-09 |
| TUBA1B | 33.97079 | 74.63679 | 1.135592 | 1.68E-11 | 1.77E-10 |
| MMP3 | 6.610199 | 61.0736 | 3.207783 | 3.15E-10 | 2.32E-09 |
| ECE1 | 11.45153 | 23.28146 | 1.023642 | 8.21E-07 | 2.75E-06 |
| IDO1 | 2.86977 | 14.43726 | 2.33079 | 4.51E-08 | 1.98E-07 |
| PDIA2 | 0.474437 | 1.593207 | 1.747645 | 0.008624 | 0.013161 |
| SLPI | 1845.444 | 684.4361 | -1.43098 | 4.07E-06 | 1.19E-05 |
| SLC1A1 | 3.109002 | 1.346908 | -1.2068 | 1.43E-09 | 9.11E-09 |
| CYP27A1 | 11.46097 | 4.943158 | -1.21322 | 1.21E-08 | 6.07E-08 |
| PFN1 | 161.6584 | 334.3568 | 1.04844 | 2.25E-12 | 2.97E-11 |
| NRG1 | 1.305616 | 4.776013 | 1.871076 | 2.57E-11 | 2.55E-10 |
| EIF4EBP1 | 30.41806 | 65.81405 | 1.113467 | 4.81E-06 | 1.39E-05 |
| TRIM21 | 7.814381 | 17.341 | 1.149984 | 7.65E-09 | 3.96E-08 |
| SLC6A2 | 0.684011 | 1.656495 | 1.276042 | 5.60E-07 | 1.94E-06 |
| CFTR | 4.022601 | 0.563532 | -2.83556 | 1.68E-15 | 8.45E-14 |
| ERBB4 | 0.965586 | 0.48068 | -1.00633 | 3.19E-11 | 3.10E-10 |
| KLF4 | 71.71557 | 33.03321 | -1.11837 | 0.000537 | 0.001049 |
| HLA-B | 231.1304 | 746.9806 | 1.692364 | 1.38E-09 | 8.87E-09 |
| ADA | 3.323867 | 10.31848 | 1.634296 | 3.39E-14 | 9.17E-13 |
| CXCL10 | 10.56438 | 123.9353 | 3.552307 | 5.39E-07 | 1.88E-06 |
| MMP13 | 0.112726 | 66.60989 | 9.206776 | 1.16E-08 | 5.89E-08 |
| ALDH9A1 | 43.92663 | 20.33385 | -1.11121 | 5.61E-14 | 1.24E-12 |
| BIRC5 | 3.563729 | 17.02972 | 2.256596 | 2.23E-17 | 3.49E-15 |
| ENO2 | 1.36532 | 8.140077 | 2.575803 | 1.26E-13 | 2.40E-12 |
| GJA1 | 53.69292 | 157.6356 | 1.553789 | 2.26E-09 | 1.36E-08 |
| CDK1 | 3.953739 | 12.08375 | 1.611779 | 4.06E-15 | 1.73E-13 |
| IRAK1 | 21.25841 | 43.7801 | 1.042241 | 1.55E-13 | 2.82E-12 |
| CCNA2 | 4.342153 | 13.60824 | 1.647998 | 8.18E-14 | 1.74E-12 |
| ALDH1A1 | 40.46847 | 10.72714 | -1.91553 | 8.41E-13 | 1.21E-11 |
| HSPB2 | 5.451047 | 1.842964 | -1.56451 | 0.000327 | 0.000666 |
| ABCC1 | 8.406161 | 19.65909 | 1.225678 | 8.00E-08 | 3.34E-07 |
| E2F1 | 1.753503 | 8.05422 | 2.199505 | 2.52E-17 | 3.49E-15 |
| CALB1 | 0.523112 | 3.657967 | 2.805851 | 4.91E-10 | 3.40E-09 |
| S100A9 | 13078.45 | 5757.848 | -1.18359 | 0.002349 | 0.003993 |
| ANK2 | 2.098976 | 0.848547 | -1.30662 | 2.19E-09 | 1.33E-08 |
| PCNA | 30.94806 | 78.93031 | 1.350731 | 1.91E-12 | 2.61E-11 |
| BMP2 | 4.485407 | 9.565714 | 1.092634 | 1.51E-06 | 4.84E-06 |
| LCN2 | 354.4006 | 111.3938 | -1.66971 | 1.08E-05 | 2.95E-05 |
| TNFSF10 | 25.71518 | 73.93939 | 1.523723 | 2.20E-07 | 8.38E-07 |
| DMD | 3.722521 | 1.511047 | -1.30073 | 2.45E-05 | 6.07E-05 |
| FCGR2A | 2.564771 | 5.953967 | 1.215021 | 6.60E-09 | 3.50E-08 |
| CSF2 | 0.482268 | 4.570882 | 3.244567 | 2.28E-14 | 7.02E-13 |
| ACTA1 | 1914.952 | 150.0503 | -3.67379 | 1.03E-06 | 3.38E-06 |
| LTF | 957.1833 | 20.82519 | -5.52239 | 9.42E-14 | 1.90E-12 |
| LPO | 20.43945 | 0.367682 | -5.79676 | 0.003203 | 0.005328 |
| ITGB1 | 22.60332 | 48.33411 | 1.096507 | 6.22E-07 | 2.13E-06 |
| SERPINA1 | 4.160745 | 9.12094 | 1.132341 | 0.001444 | 0.002558 |
| PIK3R1 | 9.855261 | 4.842531 | -1.02513 | 2.20E-05 | 5.52E-05 |
| CXCL12 | 16.24858 | 6.027268 | -1.43074 | 1.97E-06 | 6.16E-06 |
| BAK1 | 11.05201 | 27.83429 | 1.332555 | 6.95E-12 | 8.19E-11 |
| SIGMAR1 | 16.01534 | 36.40792 | 1.184798 | 1.38E-10 | 1.11E-09 |
| TGFBR1 | 5.799751 | 11.78232 | 1.022561 | 7.67E-12 | 8.76E-11 |
| MIF | 22.59211 | 46.49021 | 1.041108 | 3.11E-08 | 1.42E-07 |
| PXN | 7.705885 | 21.62925 | 1.488951 | 7.40E-13 | 1.14E-11 |
| EIF2AK2 | 3.790326 | 9.684428 | 1.353345 | 1.26E-14 | 4.29E-13 |
| CD44 | 42.75595 | 89.96976 | 1.073315 | 4.59E-10 | 3.22E-09 |
| S100A8 | 5399.856 | 2053.364 | -1.39493 | 0.000464 | 0.000917 |
| NAGS | 1.006131 | 2.647868 | 1.396013 | 4.69E-13 | 7.76E-12 |
| ADH1C | 5.474346 | 1.908197 | -1.52048 | 2.34E-11 | 2.40E-10 |
| DNMT1 | 5.421087 | 11.31784 | 1.061944 | 4.61E-12 | 5.67E-11 |
| SCN4A | 4.097048 | 0.8975 | -2.1906 | 3.53E-05 | 8.62E-05 |
| TGFB3 | 3.786879 | 7.742364 | 1.031765 | 3.01E-05 | 7.37E-05 |
| SNTA1 | 18.70647 | 8.140366 | -1.20037 | 0.019379 | 0.027599 |
| AMPD1 | 13.31841 | 1.321596 | -3.33307 | 8.48E-09 | 4.35E-08 |
| UCP3 | 3.362687 | 1.00806 | -1.73803 | 6.49E-05 | 0.000151 |
| MET | 5.301999 | 14.05076 | 1.40604 | 3.39E-11 | 3.21E-10 |
| NGB | 0.429825 | 1.115576 | 1.375969 | 6.75E-05 | 0.000155 |
| MMP1 | 33.97988 | 339.9035 | 3.322373 | 6.70E-12 | 7.98E-11 |
| STAT1 | 20.98479 | 79.78061 | 1.926694 | 1.91E-10 | 1.50E-09 |
| AOC3 | 5.106844 | 2.465375 | -1.05062 | 0.002963 | 0.004966 |
| NR3C2 | 2.78246 | 0.800405 | -1.79756 | 3.59E-12 | 4.57E-11 |
| GPT | 6.08921 | 1.091512 | -2.47993 | 1.01E-13 | 2.01E-12 |
| TFRC | 9.073931 | 25.91277 | 1.513864 | 5.18E-08 | 2.25E-07 |
| PLA2G7 | 2.391958 | 5.232135 | 1.129208 | 5.56E-10 | 3.80E-09 |
| ACHE | 5.51417 | 1.723917 | -1.67745 | 2.98E-05 | 7.33E-05 |
| CRAT | 32.71578 | 6.751953 | -2.27661 | 2.93E-06 | 8.91E-06 |
| NR4A2 | 9.249314 | 3.815652 | -1.27742 | 0.00029 | 0.0006 |
| EGR1 | 177.3057 | 80.61541 | -1.13711 | 3.80E-07 | 1.38E-06 |
| CDKN2A | 0.864669 | 9.06058 | 3.389383 | 0.000298 | 0.000614 |
| CCL5 | 8.646969 | 43.07316 | 2.316523 | 9.28E-07 | 3.09E-06 |
| ETS1 | 5.922633 | 12.60382 | 1.08955 | 1.53E-07 | 6.02E-07 |
| RAC2 | 6.225807 | 24.28021 | 1.963448 | 1.17E-13 | 2.28E-12 |
| TF | 10.6216 | 1.360225 | -2.96508 | 5.10E-14 | 1.23E-12 |
| CDK2 | 4.310023 | 9.286158 | 1.107386 | 1.76E-14 | 5.56E-13 |
| DUSP1 | 284.7327 | 130.0724 | -1.13029 | 5.86E-07 | 2.01E-06 |
| HSP90B1 | 66.47858 | 147.8742 | 1.153408 | 1.42E-13 | 2.61E-12 |
| FN1 | 4.373262 | 117.0771 | 4.742605 | 5.74E-11 | 5.13E-10 |
| TREM2 | 3.384004 | 12.36185 | 1.869091 | 1.07E-11 | 1.17E-10 |
| IL1RN | 246.9711 | 75.85875 | -1.70295 | 0.00075 | 0.001428 |
| CACNA1S | 11.10213 | 1.584838 | -2.80843 | 0.000941 | 0.001743 |
| PLAU | 9.134373 | 95.32574 | 3.383488 | 7.75E-17 | 7.15E-15 |
| CALR | 162.663 | 337.6027 | 1.05344 | 2.21E-15 | 9.78E-14 |
| MGST1 | 9.737802 | 4.537142 | -1.10181 | 3.33E-07 | 1.23E-06 |
| TTN | 13.50344 | 2.182537 | -2.62925 | 0.001942 | 0.003362 |
| ARG1 | 4.839602 | 2.353856 | -1.03986 | 0.011605 | 0.017122 |
| SLC25A4 | 34.25977 | 4.525181 | -2.92047 | 3.83E-10 | 2.76E-09 |
| HP | 1.802737 | 0.798683 | -1.17449 | 1.65E-09 | 1.02E-08 |
| PRKAA2 | 3.281439 | 1.021707 | -1.68335 | 2.40E-10 | 1.83E-09 |
| LOX | 4.009085 | 9.942474 | 1.310332 | 2.49E-06 | 7.62E-06 |
| CRYAB | 178.5596 | 32.37724 | -2.46335 | 2.74E-09 | 1.60E-08 |
| EGFR | 10.23491 | 32.09551 | 1.648873 | 6.54E-07 | 2.22E-06 |
| CTLA4 | 1.132174 | 2.424919 | 1.098841 | 2.69E-09 | 1.59E-08 |
| IL1A | 3.272587 | 20.95421 | 2.678737 | 1.18E-07 | 4.73E-07 |
| SPP1 | 13.80991 | 88.65798 | 2.682546 | 4.89E-07 | 1.71E-06 |
| AGT | 6.606007 | 2.239808 | -1.5604 | 4.41E-07 | 1.56E-06 |
| CLU | 101.2128 | 15.02926 | -2.75155 | 8.44E-14 | 1.76E-12 |
| CDKN3 | 2.913855 | 9.331708 | 1.679212 | 1.55E-17 | 2.85E-15 |
| HBB | 75.66199 | 10.47912 | -2.85205 | 0.005398 | 0.00868 |
| SERPINE1 | 6.569295 | 112.3943 | 4.096687 | 1.32E-14 | 4.29E-13 |
| TXNRD1 | 7.169331 | 15.21461 | 1.085547 | 0.00029 | 0.0006 |
| TRDN | 28.25706 | 2.901923 | -3.28353 | 9.07E-05 | 0.000204 |
| CASQ2 | 13.37613 | 6.27787 | -1.09131 | 0.002149 | 0.00368 |
| SHC1 | 15.68979 | 38.05033 | 1.278083 | 1.26E-14 | 4.29E-13 |
| TH | 0.531807 | 1.435599 | 1.432679 | 9.26E-08 | 3.83E-07 |
| NUDT1 | 5.490736 | 13.61643 | 1.310277 | 2.11E-12 | 2.82E-11 |
| SLC2A1 | 29.91489 | 188.5246 | 2.655817 | 2.95E-14 | 8.61E-13 |
| SOD3 | 12.41217 | 4.757518 | -1.38347 | 2.10E-07 | 8.02E-07 |
| MB | 588.2759 | 34.45348 | -4.09377 | 4.93E-08 | 2.16E-07 |
| MMP9 | 2.188944 | 70.41943 | 5.007667 | 9.06E-16 | 4.78E-14 |
| EGF | 2.011672 | 0.898661 | -1.16255 | 4.57E-06 | 1.32E-05 |
| MYH7 | 306.7088 | 14.08465 | -4.44467 | 3.09E-08 | 1.42E-07 |
| MAOB | 12.0304 | 3.364966 | -1.83802 | 7.40E-13 | 1.14E-11 |
| GPX3 | 104.2616 | 19.68579 | -2.40498 | 1.75E-13 | 3.07E-12 |
| PPARG | 2.984391 | 0.966112 | -1.62717 | 4.22E-14 | 1.07E-12 |
| ADIPOQ | 5.421232 | 0.614003 | -3.1423 | 3.54E-18 | 9.80E-16 |
| ABCD1 | 2.34332 | 5.23379 | 1.159302 | 4.10E-10 | 2.89E-09 |
| NCF2 | 4.293484 | 11.36883 | 1.404863 | 3.77E-12 | 4.70E-11 |
| PRNP | 40.66012 | 129.9423 | 1.676185 | 9.23E-14 | 1.89E-12 |
| FOS | 261.2556 | 116.1887 | -1.16899 | 1.74E-07 | 6.74E-07 |
| HIF1A | 26.06908 | 56.74269 | 1.122095 | 6.00E-09 | 3.20E-08 |
| PPARGC1A | 2.804629 | 0.885184 | -1.66376 | 3.35E-11 | 3.21E-10 |
| CAV1 | 14.54135 | 91.34721 | 2.651199 | 7.39E-12 | 8.53E-11 |
| OLR1 | 1.393674 | 3.749512 | 1.42781 | 2.28E-06 | 7.06E-06 |
| ICAM1 | 9.790891 | 20.06517 | 1.035181 | 2.65E-05 | 6.55E-05 |
| CP | 6.50485 | 2.58363 | -1.33212 | 8.65E-08 | 3.59E-07 |
| CAV3 | 6.094297 | 1.337783 | -2.18762 | 0.004485 | 0.007265 |
| TYR | 1.365142 | 0.551096 | -1.30867 | 1.91E-05 | 4.87E-05 |
| DDIT3 | 5.697845 | 11.4386 | 1.005422 | 2.00E-05 | 5.07E-05 |
| ETFDH | 8.709907 | 4.30721 | -1.0159 | 2.65E-14 | 7.94E-13 |
| MAPT | 2.704117 | 0.805543 | -1.74712 | 1.84E-12 | 2.58E-11 |
| VEGFA | 4.324531 | 9.026029 | 1.061548 | 3.17E-08 | 1.45E-07 |
| CCL2 | 32.07075 | 10.83992 | -1.5649 | 1.29E-07 | 5.15E-07 |
| RYR1 | 22.79627 | 4.469697 | -2.35055 | 0.028558 | 0.039162 |
| PTGS2 | 3.068426 | 10.91355 | 1.83055 | 0.002639 | 0.004457 |
| TGFB1 | 8.127367 | 39.07776 | 2.265488 | 1.02E-18 | 5.63E-16 |
| CXCL8 | 11.84115 | 41.80178 | 1.819756 | 7.30E-05 | 0.000166 |
| HADH | 23.38079 | 11.6037 | -1.01074 | 3.73E-11 | 3.47E-10 |
| ACADM | 14.14991 | 6.596441 | -1.10103 | 7.04E-07 | 2.37E-06 |
| IL1B | 4.02048 | 12.76362 | 1.666598 | 5.46E-05 | 0.00013 |
| APP | 55.19566 | 124.3084 | 1.171297 | 4.23E-09 | 2.34E-08 |
| MPO | 1.616325 | 0.525685 | -1.62045 | 0.007408 | 0.011498 |
| NOS1 | 2.568558 | 0.933158 | -1.46077 | 4.83E-07 | 1.71E-06 |

ORGs: oxidative stress-related genes; OSCC, oral squamous cell carcinoma; N-Mean: the mean value of gene expression in normal tissues; T-Mean: the mean value of gene expression in OSCC tissues; FC, fold change; FDR, false discovery rate.
