# Supplementary material for: An oxidative stress-related prognostic signature for indicating the immune status of oral squamous cell carcinoma and guiding clinical treatment
Source: Front Genet. 2022 Sep 23;13:977902. doi: 10.3389/fgene.2022.977902 (PMC9538189; doi:10.3389/fgene.2022.977902)
Supplement: Supplementary file 10 [file Table4.DOCX]

https://www.jianguoyun.com/p/DUhGKiQQg7HAChjezrkEIAA
